# Supplementary material for: Smoking, DNA Methylation, and Lung Function: a Mendelian Randomization Analysis to Investigate Causal Pathways
Source: Am J Hum Genet. 2020 Feb 20;106(3):315–26. doi: 10.1016/j.ajhg.2020.01.015 (PMC7058834; doi:10.1016/j.ajhg.2020.01.015)
Supplement: Document S1. Figures S1–S9 [file mmc1.pdf]

The American Journal of Human Genetics, Volume *106*

Supplemental Data

Smoking, DNA Methylation, and Lung Function: a Mendelian Randomization Analysis to Investigate Causal Pathways

Emily Jamieson, Roxanna Korologou-Linden, Robyn E. Wootton, Anna L. Guyatt, Thomas Battram, Kimberley Burrows, Tom R. Gaunt, Martin D. Tobin, Marcus Munafò, George Davey Smith, Kate Tilling, Caroline Relton, Tom G. Richardson, and Rebecca C. Richmond

Supplementary figures

Figure S1. Quantile-quantile plot of the observed vs expected p-values of the associations between smoking-associated DNA methylation and lung function (FEV_1).

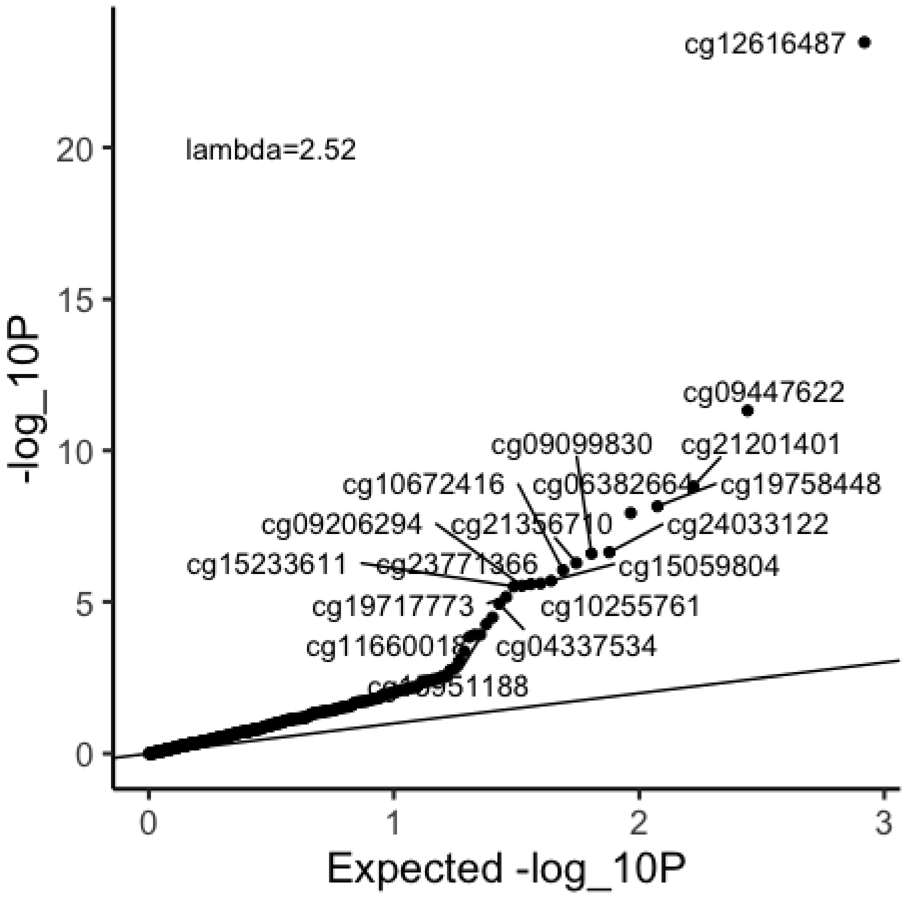

The line represents the null hypothesis of no association with lung function. Deviation from the expected distribution of P-values is evident with a lambda of 2.52.

Figure S2. Results of MR analysis of the effect of smoking-associated DNA methylation on lung function (FEV1) stratified by smoking status.

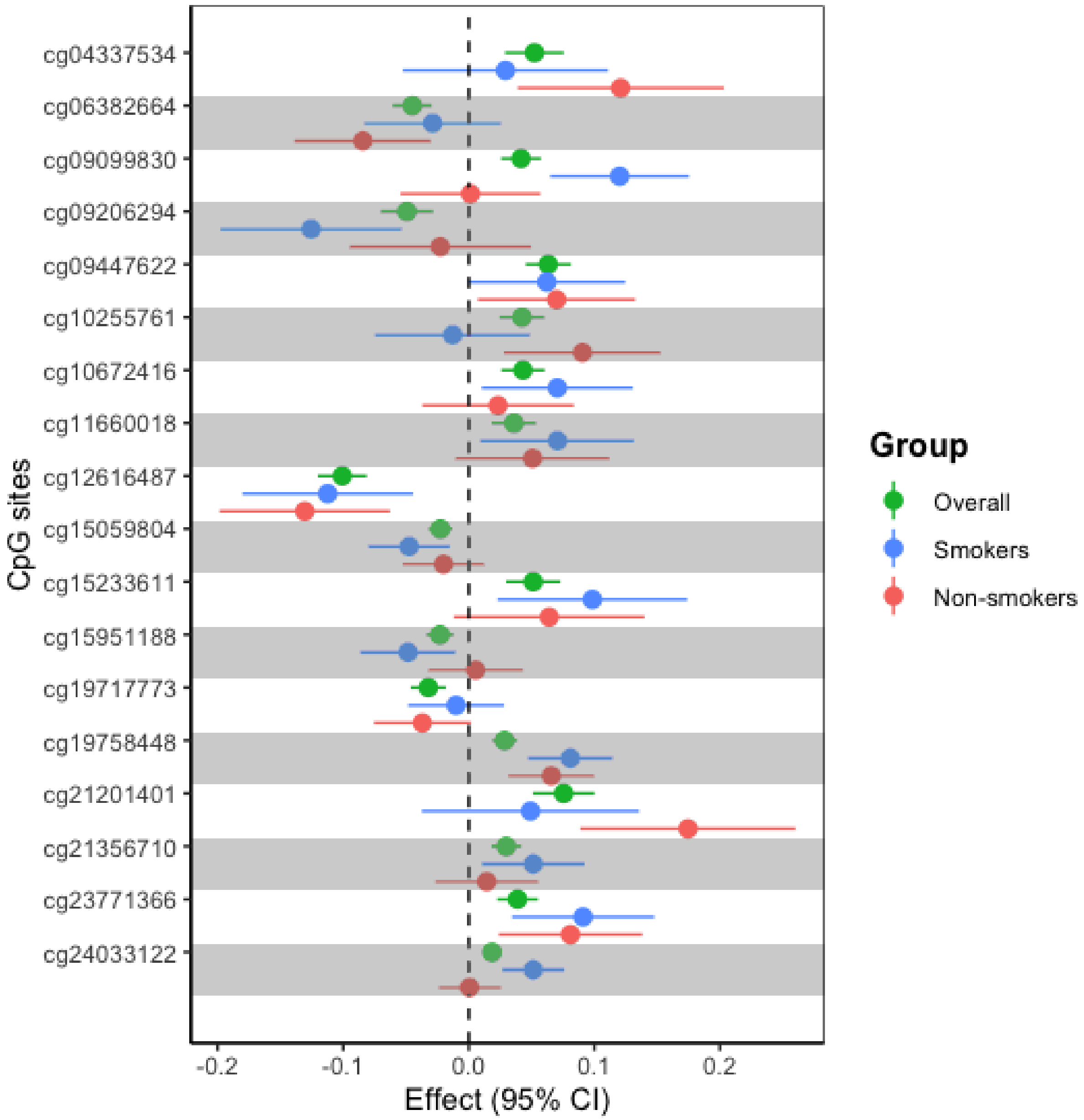

Effect sizes and 95% confidence intervals (CI) for each of the top eighteen CpG sites are shown for the overall UK Biobank sample in green, and the smoking and non-smoking UK BiLEVE samples in blue and red, respectively.

Figure S3. Results of MR analysis of the effect of smoking-associated DNA methylation on lung function (FEV1, FVC and FEV1/FVC)

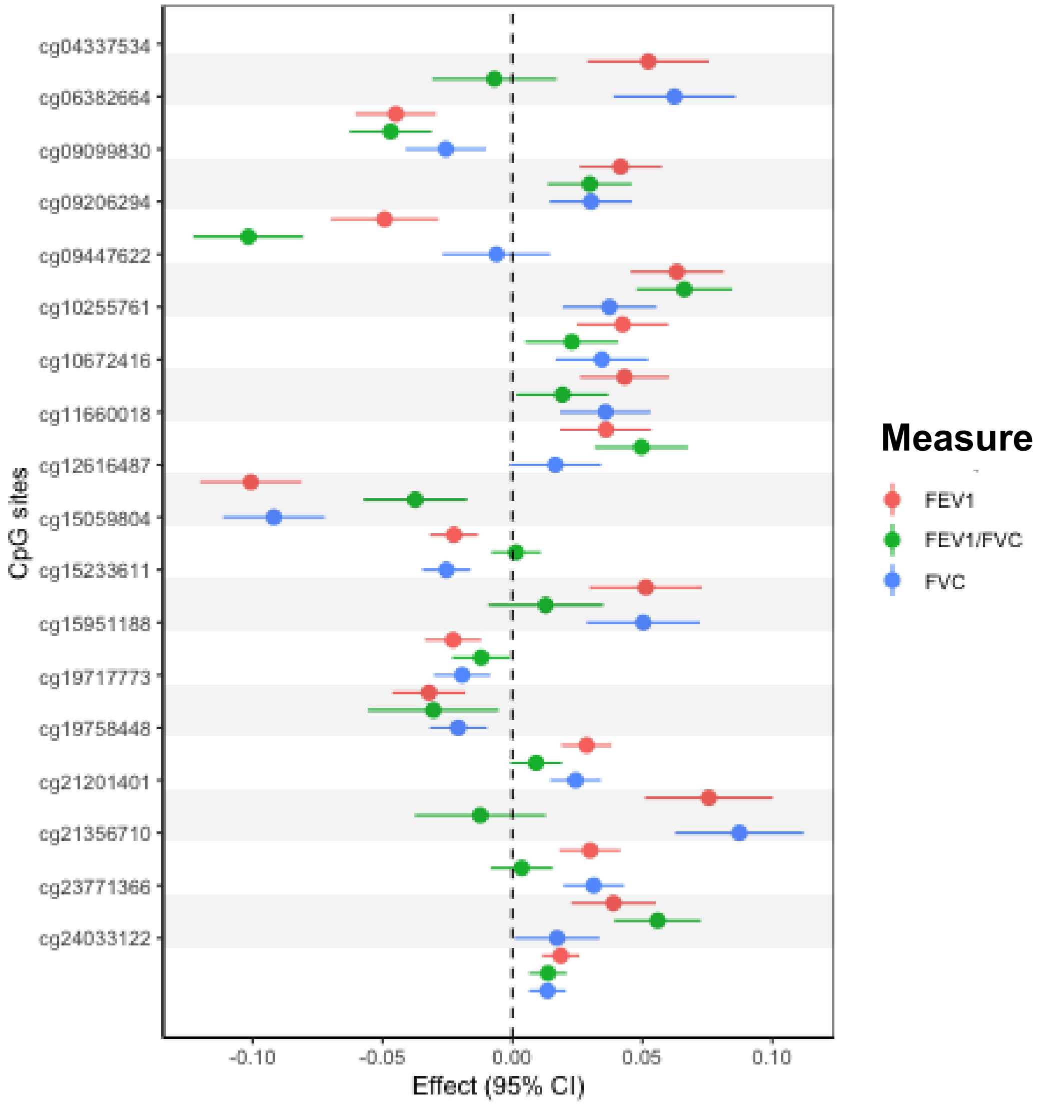

Effect sizes and 95% confidence intervals (CI) for each of the top eighteen CpG sites are shown for FEV1 in red, FEV1/FVC in green and FVC in blue.

Figure S4. Results of MR analysis of the effect of smoking-associated DNA methylation on lung disease (asthma and COPD)

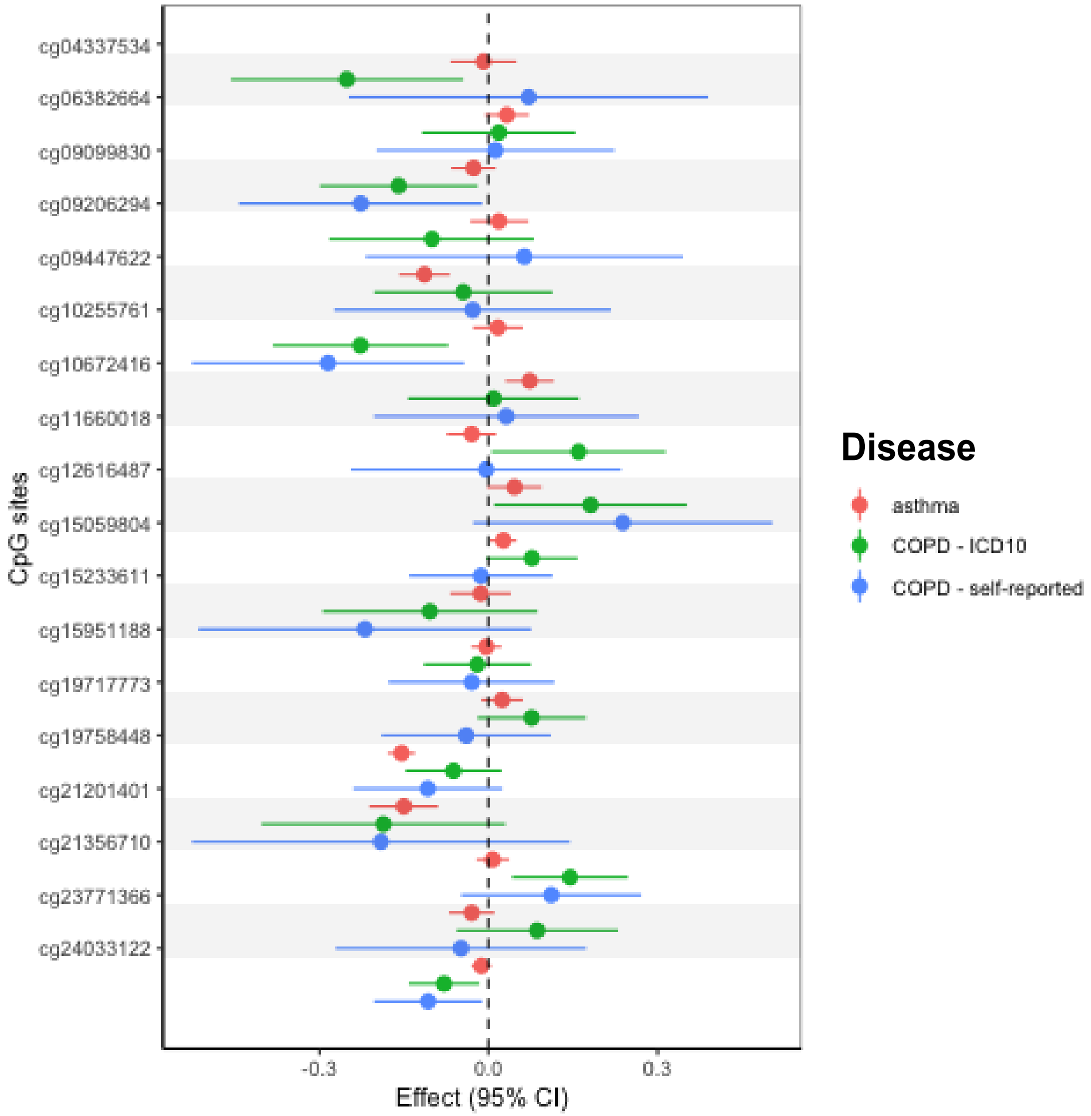

Effect sizes and 95% confidence intervals (CI) for each of the top eighteen CpG sites are shown for asthma in red, COPD obtained from ICD-10 codes in green and COPD based on self-report in blue

Figure S5. Comparison of observational and Mendelian randomization effect estimates of smoking on DNA methylation at smoking-related CpG sites*

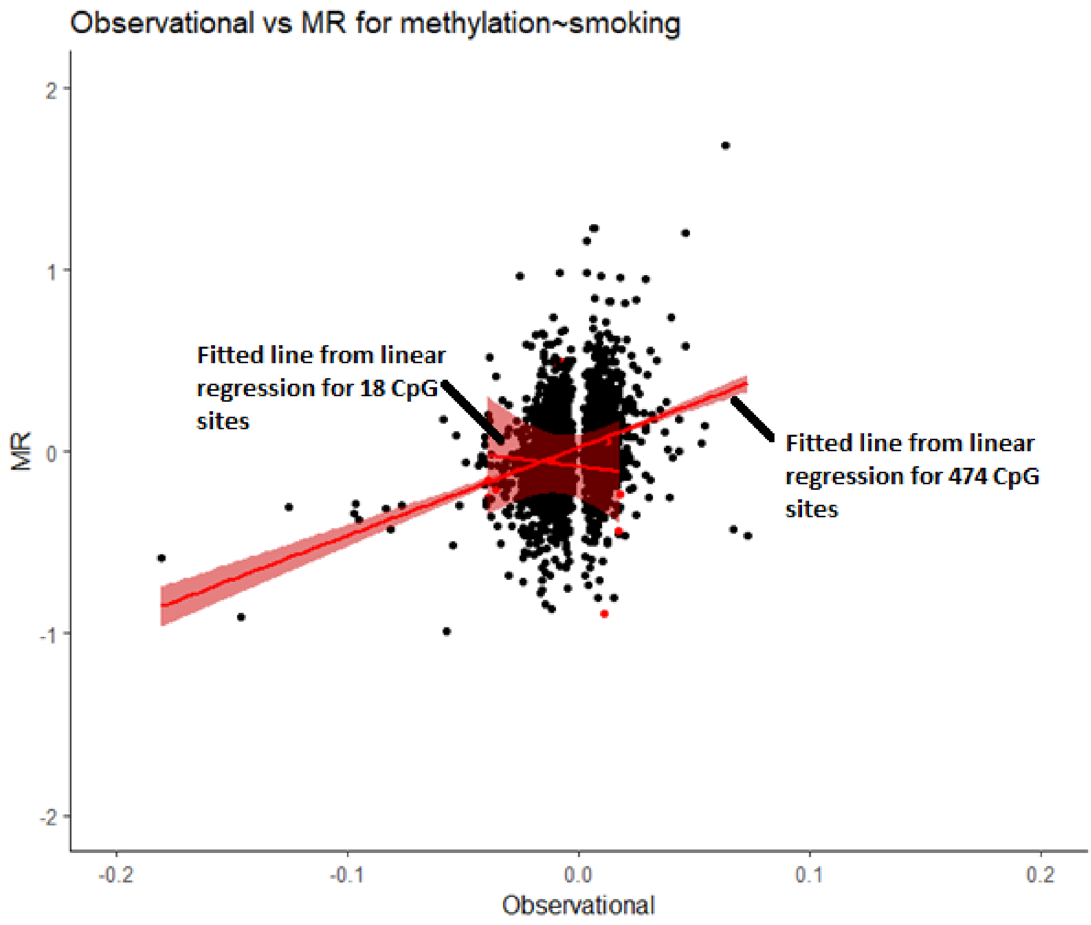

*Obtained from epigenome-wide association study of smoking by Joehanes et al, 2016 [1]

1 Joehanes, R. *et al.* Epigenetic Signatures of Cigarette Smoking. *Circ.-Cardiovasc. Genet.* **9**, 436-447, doi:10.1161/circgenetics.116.001506 (2016).

Figure S6. Comparison of MR analyses accounting for correlated mQTLs with those using only independent mQTLs

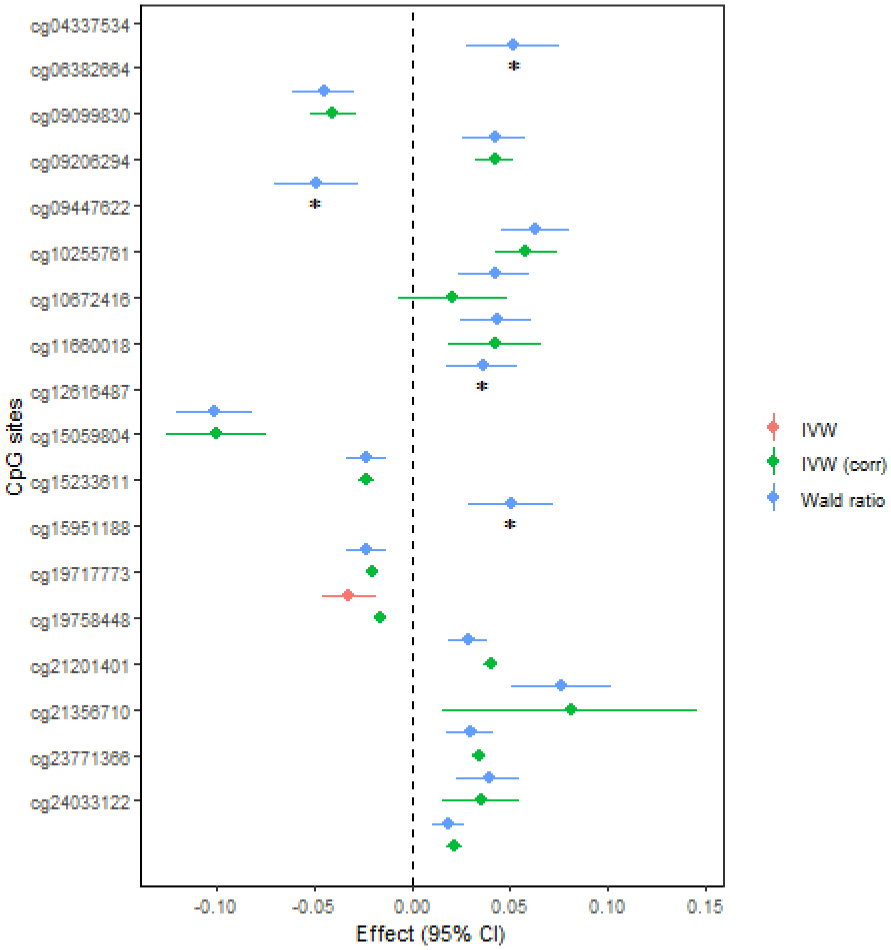

Effect sizes and 95% confidence intervals (CI) for each of the top eighteen CpG sites are shown from MR using only independent mQTLs (IVW in red and Wald ratio in blue), and accounting for correlated mQTLs (IVW (corr) in green). CpG sites where there was only one mQTL in the correlation analysis are indicated with an asterisk.

Figure S7. Egger regression plots for MR analysis accounting for correlated mQTLs

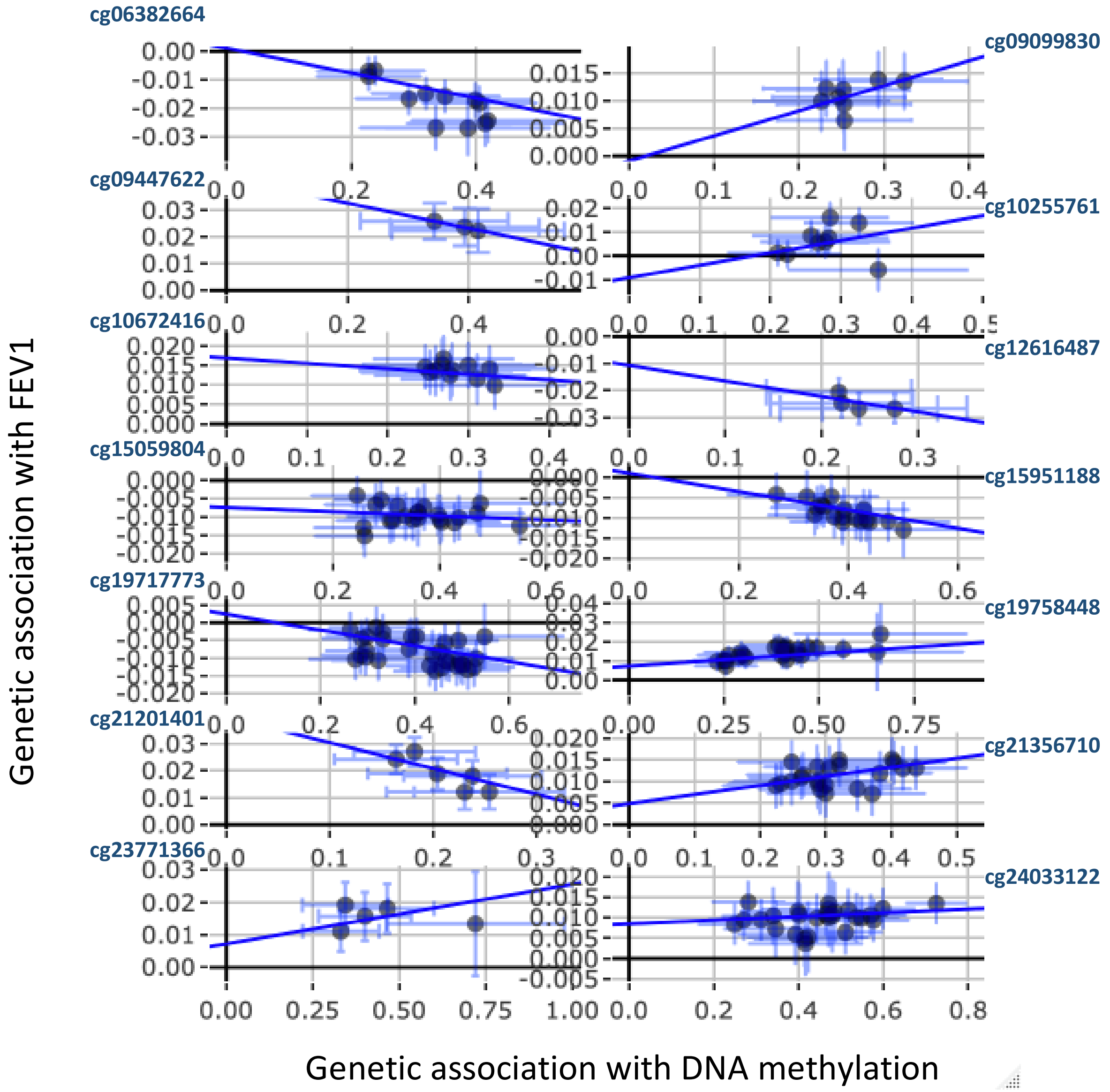

Where MR Egger regression line intercept deviates from the origin, this is indicative of directional pleiotropy

Figure S8. Comparison of MR analyses accounting for correlated mQTLs

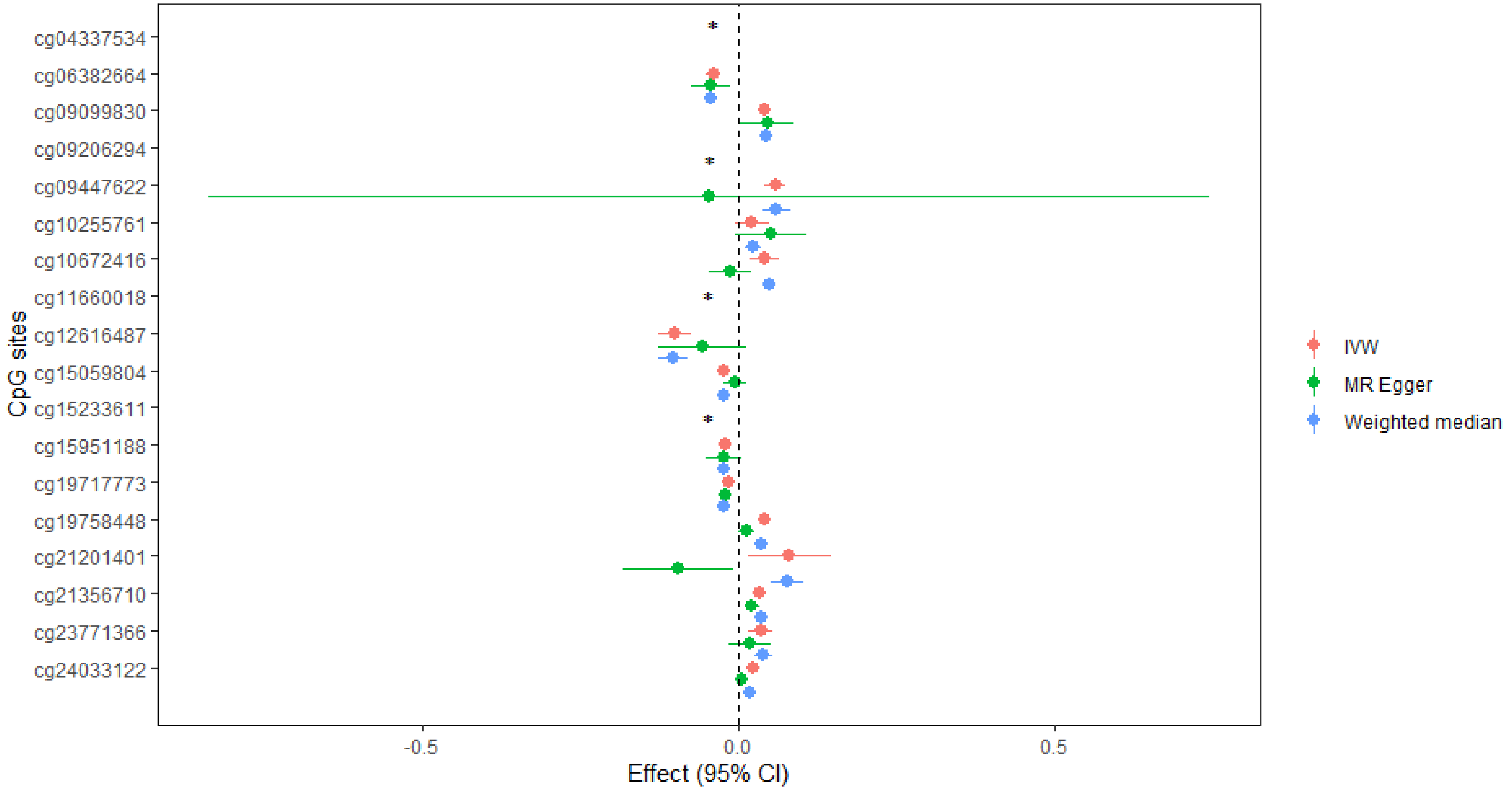

Effect sizes and 95% confidence intervals (CI) for each of the top eighteen CpG sites are shown from MR IVW in red, MR Egger in green and the weighted median approach in blue. Sites where there were too few mQTLs to perform the analysis are indicated with an asterisk.

Figure S9. Locus zoom plots for multiple-trait colocalization analysis

a) cg15951188

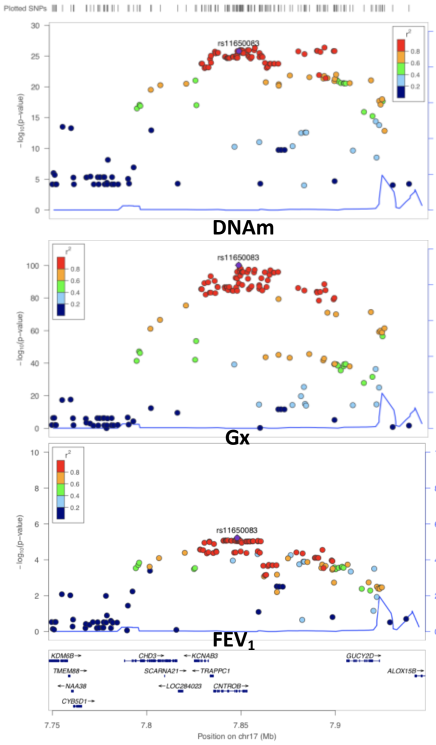

b) cg12616487

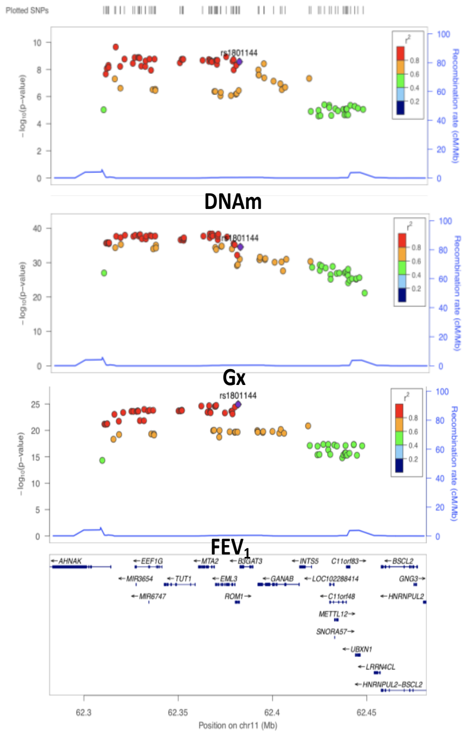

c) cg21201401

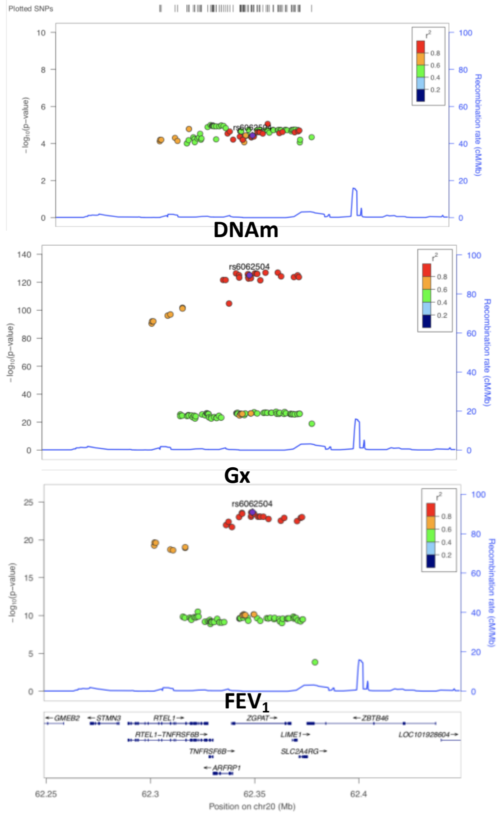

d) cg15233611

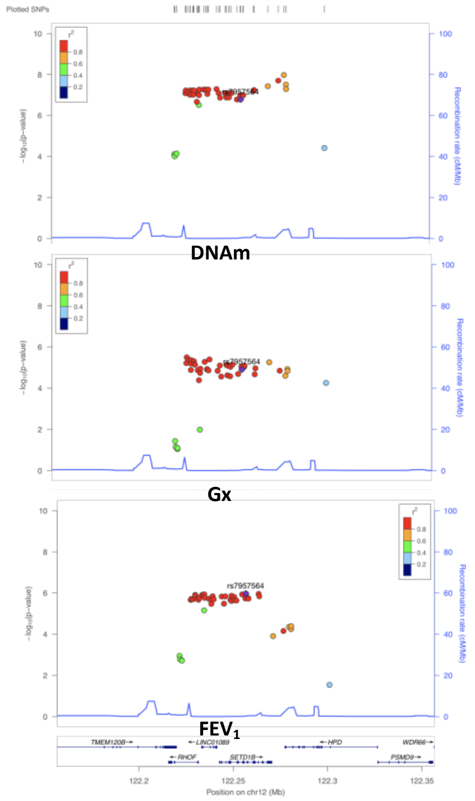

e) cg04337534

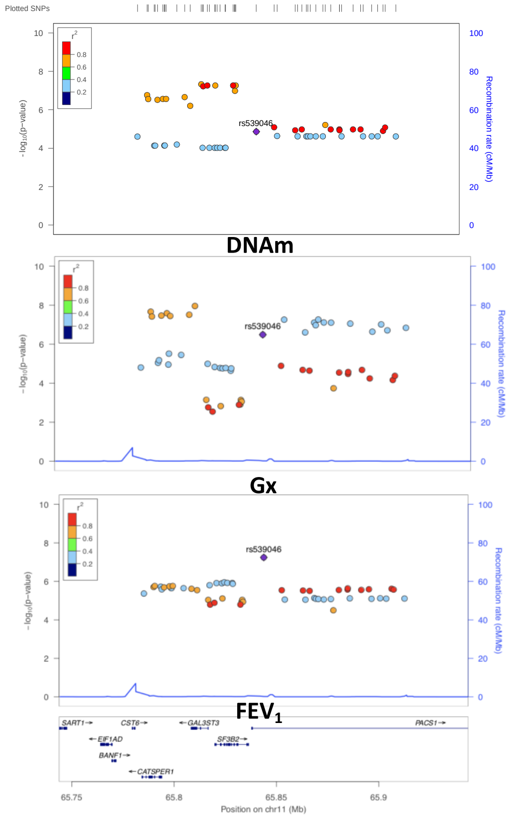

f) cg09447622

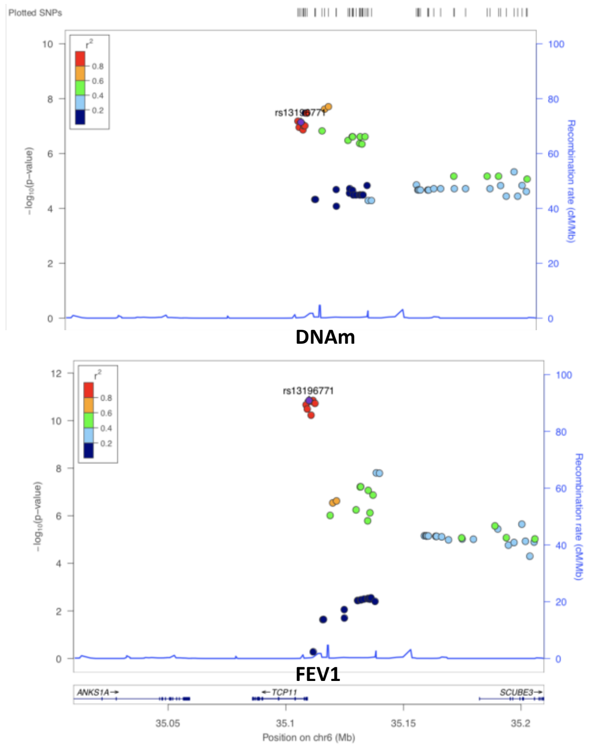

g) cg19758448

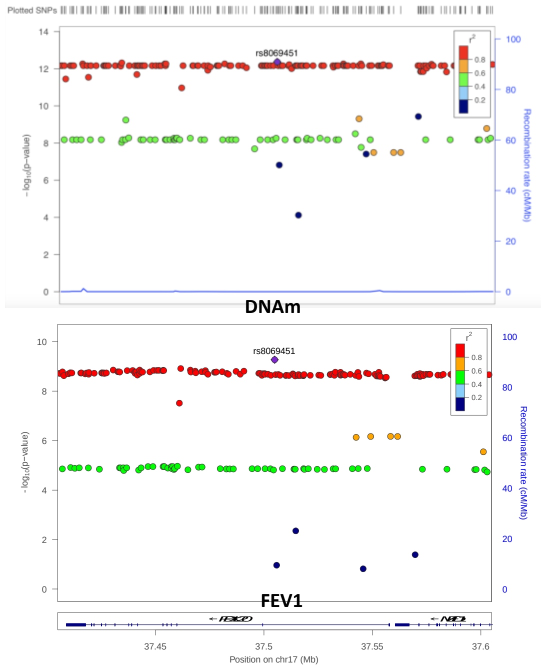

h) cg09206294

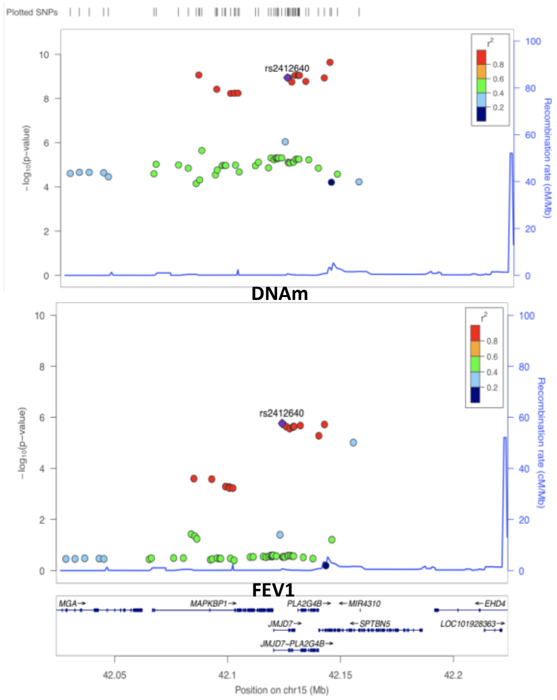

i) cg21356710

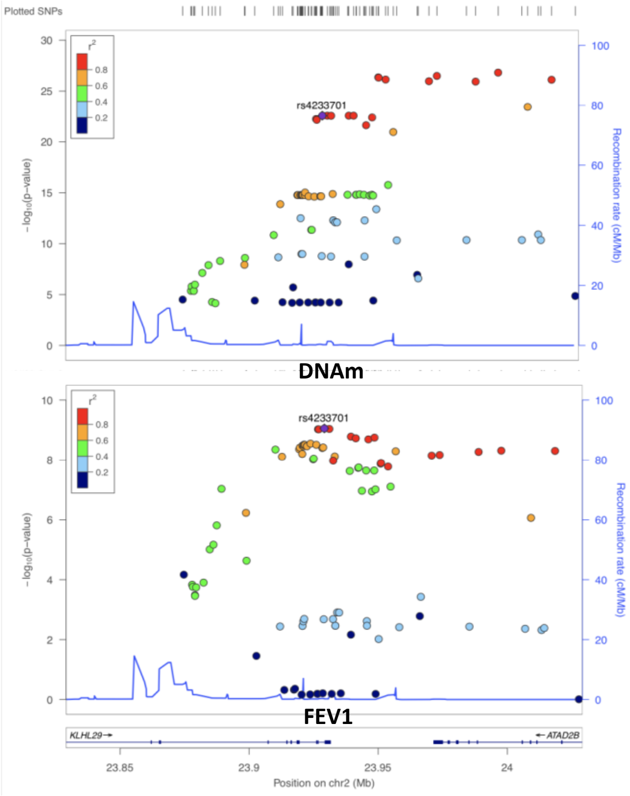

DNAm = DNA methylation; Gx = Gene expression; FEV1 = forced expiratory volume in 1 second
