# Supplementary material for: Smoking, DNA Methylation, and Lung Function: a Mendelian Randomization Analysis to Investigate Causal Pathways
Source: Am J Hum Genet. 2020 Feb 20;106(3):315–26. doi: 10.1016/j.ajhg.2020.01.015 (PMC7058834; doi:10.1016/j.ajhg.2020.01.015)
Supplement: Document S3. Article plus Supplemental Information [file mmc3.pdf]

Smoking, DNA Methylation, and Lung Function: a Mendelian Randomization Analysis to Investigate Causal Pathways

Emily Jamieson,[1,2] Roxanna Korologou-Linden,[1,2] Robyn E. Wootton,[1,3,4] Anna L. Guyatt,[5] Thomas Battram,[1,2] Kimberley Burrows,[1,2] Tom R. Gaunt,[1,2,4] Martin D. Tobin,[5] Marcus Munafò,[1,3,4] George Davey Smith,[1,2,4] Kate Tilling,[1,2] Caroline Relton,[1,2] Tom G. Richardson,[1,2] and Rebecca C. Richmond[1,2,*]

Whether smoking-associated DNA methylation has a causal effect on lung function has not been thoroughly evaluated. We first investigated the causal effects of 474 smoking-associated CpGs on forced expiratory volume in 1 s (FEV_1) in UK Biobank (n = 321,047) by using two-sample Mendelian randomization (MR) and then replicated this investigation in the SpiroMeta Consortium (n = 79,055). Second, we used two-step MR to investigate whether DNA methylation mediates the effect of smoking on FEV_1. Lastly, we evaluated the presence of horizontal pleiotropy and assessed whether there is any evidence for shared causal genetic variants between lung function, DNA methylation, and gene expression by using a multiple-trait colocalization ("moloc") framework. We found evidence of a possible causal effect for DNA methylation on FEV_1 at 18 CpGs ($p < 1.2 \times 10^{-4}$). Replication analysis supported a causal effect at three CpGs (cg21201401 [*LIME1* and *ZGPAT*], cg19758448 [*PGAP3*], and cg12616487 [*EML3* and *AHNAK*] [$p < 0.0028$]). DNA methylation did not clearly mediate the effect of smoking on FEV_1, although DNA methylation at some sites might influence lung function via effects on smoking. By using "moloc", we found evidence of shared causal variants between lung function, gene expression, and DNA methylation. These findings highlight potential therapeutic targets for improving lung function and possibly smoking cessation, although larger, tissue-specific datasets are required to confirm these results.

Introduction

Cigarette smoking is a major risk factor for lung disease, which is often preceded by a rapid decline in lung function.[1] Studies have shown a strong causal role of smoking in relation to lung-function decline, which can be measured by forced expiratory volume in 1 s (FEV_1).[2] Exploring the mechanistic pathways leading to decreased lung function in smokers could highlight targets for therapeutic intervention.

One mechanism that might mediate the association between smoking and decreased lung function is altered DNA methylation patterns. Smoking is associated with substantial changes to methylation levels at many loci across the genome.[3] For example, hypomethylation at the CpG site cg05575921 in intron 3 of the aryl hydrocarbon receptor repressor (*AHRR*) gene is strongly associated with both the current and past smoking behavior of an individual,[3,4] and it has recently been suggested to mediate a proportion of the effect of smoking on decreased lung function.[5] However, it is not clear that this association represents a true causal pathway.[6] Furthermore, DNA methylation at other CpG sites related to lung function might also serve as a potential mediator on the pathway from smoking.[7,8]

Mendelian randomization (MR) is a method that researchers can use to assess the causality of a modifiable exposure on an outcome[9] by using genetic variants robustly associated with the exposure as proxies. Because genetic variants are effectively randomized at conception, they are unlikely to be influenced by confounding factors that might otherwise bias associations in observational analysis. In the context of methylation, MR is facilitated by genetic variants, known as mQTLs (methylation quantitative trait loci), that are found to be strongly associated with DNA methylation.[10]

Among the many extensions of the basic MR principle[11] is the two-step method, which aims to assess whether an intermediate factor has a causal role in the mediating pathway between the exposure and the outcome.[12] A further extension is the two-sample framework, which allows the exposure and outcome data to come from two independent datasets so that the effect of the genetic variant on the exposure and outcome can be estimated separately.[13] Both approaches are particularly advantageous for epigenetic studies: two-step MR can be used so that DNA methylation might serve as an intermediate between a particular exposure and outcome, and two-sample MR can be used because DNA methylation datasets are unlikely to include the relevant exposure and/or outcome data of interest. Researchers can use these methods to evaluate the causal role of DNA methylation at a large number of CpG sites; they can also use these methods within a

[1]Medical Research Council Integrative Epidemiology Unit at the University of Bristol, University of Bristol, Oakfield House, Oakfield Grove, Bristol BS8 2BN, UK; [2]Population Health Sciences, Bristol Medical School, University of Bristol, Oakfield House, Oakfield Grove, Bristol BS8 2BN, UK; [3]School of Psychological Science, University of Bristol, 12a Priory Road, Bristol BS8 1TU, UK; [4]National Institute for Health Research Bristol Biomedical Research Centre, University Hospitals Bristol National Health Service Foundation Trust and University of Bristol, Bristol, UK; [5]Department of Health Sciences, University of Leicester, University Road, Leicester LE1 7RH, UK
*Correspondence: rebecca.richmond@bristol.ac.uk
https://doi.org/10.1016/j.ajhg.2020.01.015.
© 2020 The Authors. This is an open access article under the CC BY license (http://creativecommons.org/licenses/by/4.0/).

mediation framework to determine whether DNA methylation mediates the effect of an exposure and outcome.[12,14]

A key violation of the MR approach is horizontal pleiotropy, whereby a genetic variant used to proxy a modifiable exposure is associated with the outcome through pathways not involving the exposure. Various sensitivity analyses exist for investigating horizontal pleiotropy in MR analysis,[15] which can also be applied to assessing the validity of mQTLs as genetic proxies for DNA methylation and can be complemented by colocalization approaches[16] that help to evaluate whether the mQTL is responsible for effects on both DNA methylation and the outcome.[17] Furthermore, multiple-trait colocalization ("moloc") can also be used for determining whether variation in DNA methylation levels at putatively causal CpG sites might influence traits via changes in the expression of nearby genes.[18] Such approaches can be integrated into an analytical pipeline that can be used for highlighting and prioritizing molecular pathways for further intervention.[19]

We first aimed to search for a causal effect of methylation at smoking-associated CpG sites on FEV_1 in the UK Biobank by using two-sample MR, and we replicated this search in the SpiroMeta Consortium.[20] Second, we investigated whether DNA methylation mediates the effect of smoking on FEV_1. Lastly, we evaluated the presence of horizontal pleiotropy and also assessed whether there is any evidence for shared causal genetic variants between lung function, DNA methylation, and gene expression by using a "moloc" framework.

Material and Methods

mQTL Identification: The Accessible Resource for Integrated Epigenomic Studies in the Avon Longitudinal Study of Parents and Children

The Avon Longitudinal Study of Parents and Children (ALSPAC) is a large, prospective cohort study based in the southwest of England. A total of 14,541 pregnant women who were residing in Avon, UK, and had expected dates of delivery from April 1, 1991 to December 31, 1992 were recruited, and detailed information has been collected on these women and their offspring at regular intervals.[21,22] The study website contains details of all the data that are available through a fully searchable data dictionary. Written informed consent has been obtained for all ALSPAC participants. Ethical approval for the study was obtained from the ALSPAC Ethics and Law Committee and the local research ethics committees.

As part of the Accessible Resource for Integrated Epigenomics Studies (ARIES) project,[10,23] the Illumina Infinium HumanMethylation450 (HM450) BeadChip was used for generating epigenetic data on cord blood and peripheral blood samples from 1,018 mother-offspring pairs in the ALSPAC cohort at five time points (birth, childhood, adolescence, the antenatal period, and middle age). The ARIES participants were previously genotyped as part of the larger ALSPAC study, and quality control, cleaning, and imputation were performed at the cohort level as described previously.[10]

Matrix eQTL software[24] was used for preliminary association analysis of SNPs with CpG sites in the HM450 array; further multivariable linear regression analysis was run in PLINK1.07,[25] and genome-wide complex trait analysis (GCTA) was performed[26] as previously described[10] so that conditionally independent mQTLs could be determined. Associations with $p < 1 \times 10^{-7}$ were selected for this analysis via a publicly available online catalog.[10] For this analysis, we only considered those mQTLs identified in the middle-age time point among women in ARIES.

Genome-wide Association of Forced Expiratory Volume and Lifetime Smoking Behavior: UK Biobank

We used genetic association data from individuals in the UK Biobank. The UK Biobank study is a large population-based cohort of 502,682 individuals who were recruited at ages 37–73 years across the UK between 2006 and 2010; the study includes extensive health and lifestyle questionnaire data (including smoking behavior), physical measures (including spirometry), and DNA samples. The study protocol is available online, and more details have been published elsewhere.[27] The UK Biobank study was approved by the North West Multi-Centre Research Ethics Committee (reference number 06/MRE08/65), and at recruitment, all participants gave informed consent to participate in the UK Biobank and be followed-up with.

Participants were genotyped with either the Affymetrix UK BiLEVE Axiom Array or the Affymetrix UK Biobank Axiom Array. Details of how the genotype data were cleaned, imputed, and released to the scientific community are detailed elsewhere.[28] Summary-level genetic association statistics for FEV_1 were obtained from a recent genome-wide association study (GWAS) of FEV_1 (covariate adjusted and inverse-normal rank transformed) in the UK Biobank ($n = 321,047$)[20] and, for lifetime smoking behavior, from a GWAS of a comprehensive smoking index metric derived from data on smoking duration, heaviness, and cessation in UK Biobank participants ($n = 462,690$)[29].

Two-Sample MR: ARIES and UK Biobank

To assess the causal effect of DNA methylation at smoking-related CpG sites on lung function, we conducted two-sample MR.[13] In this approach, information on the SNP-exposure (here, DNA methylation) and SNP-outcome (here, lung function [FEV_1]) effects are derived from genome-wide association analysis conducted in separate studies with the "TwoSampleMR" package in R[15].

For the smoking-related CpG sites that could be proxied by mQTLs, we looked up the identified mQTLs in the lung function GWAS summary data from the UK Biobank. We extracted the following summary data for each SNP: the effect estimate, along with its standard error (SE), for lung function per copy of the effect allele, the reference allele, and the effect allele along with its frequency. We combined information on the SNP-lung function associations from the UK Biobank with information on the SNP-methylation associations from ARIES in order to perform the Mendelian randomization analysis described below.

For each SNP, we calculated the change in FEV_1 per standard deviation (SD) increase in methylation by the formula $\beta GD/\beta GP$ (also known as a Wald ratio), where βGD is the SD change in volume of air exhaled in 1 s per copy of the effect allele and where βGP is the SD increase in methylation per copy of the effect allele. SEs of the Wald ratios were approximated by the delta method.[30] Where multiple conditionally independent mQTLs were available for the same CpG site, we combined these in a fixed effects meta-analysis after weighting each ratio estimate by the inverse

variance of their associations with the outcome (inverse-variance weighted [IVW] approach). For all downstream analyses, we proceeded with those CpG sites where the effect of DNA methylation on FEV_1 surpassed Bonferroni correction in this main analysis.

Replication: SpiroMeta

We attempted to replicate the findings regarding the causal effect of DNA methylation by using an independent second sample for the two-sample MR approach. For this, we used data available on genetic variants and lung function (FEV_1) in 79,055 individuals of European ancestry from 22 studies, combined in a meta-analysis by the SpiroMeta Consortium.[20]

Stratification: UK BiLEVE

To investigate the extent to which the genetically predicted effects of DNA methylation on lung function are modified by smoking status, we conducted an MR analysis stratified by smoking status. For this, GWAS of FEV_1 has been undertaken in 48,931 individuals from the UK BiLEVE study, involving a subset of UK Biobank participants who were selected from the extremes of the lung-function distribution (extremely low, near average, and extremely high) and by smoking status (never versus heavy smokers [mean of 35 pack-years of smoking, where 1 pack-year is equal to smoking 20 cigarettes (1 pack) per day for 1 year]).[31,32] Genotyping was undertaken with the Affymetrix Axiom UK BiLEVE array for 24,457 smokers and 24,474 non-smokers in the UK BiLEVE study.

Causal Effects of DNA Methylation on Other Lung-Function-Related Traits

We assessed consistency of the causal effects observed for FEV_1 in relation to a number of other lung-function-related traits by using summary statistics from a UK Biobank GWAS[20] of forced vital capacity (FVC) ($n = 321,047$) and FEV_1/FVC ratio ($n = 321,047$), as well as from other UK Biobank GWAS[33] of self-reported asthma ($n = 53,598$ cases, 409,335 controls), self-reported chronic obstructive pulmonary disease (COPD) ($n = 1,605$ cases, 461,328 controls), and COPD derived from ICD-10 codes ($n = 3,871$ cases, 459,139 controls).

DNA Methylation and Lung Function: Direction of Causality

Where there was evidence that DNA methylation might have a causal effect on lung function, we evaluated the possibility of reverse causation, whereby a SNP used as a proxy for DNA methylation has its primary effect through lung function rather than through DNA methylation. For this, we performed the MR Steiger test,[34] implemented in the "TwoSampleMR" package[15] with the previously outlined summary GWAS data from ARIES and the UK Biobank, to determine the likely direction of effect.

Furthermore, we conducted the reciprocal MR at these CpG sites to appraise the causal effect of lung function (FEV_1) on DNA methylation. For this, we assessed associations between 221 SNPs with $p < 5 \times 10^{-8}$ from the UK Biobank GWAS of FEV_1 and DNA methylation at the CpG sites of interest identified in the middle-age time point among women in ARIES. Because only associations with $p < 1 \times 10^{-7}$ were available in the publicly available online catalog, we used PLINK1.07[10] to perform exact linear regression of methylation beta-values at each CpG site on SNP genotypes and also adjusted the model for age, sex, top ten ancestry principal components, bisulphite conversion batch, and estimated white blood cell counts.

Smoking Behavior and DNA Methylation: Direction of Causality

We also performed bidirectional MR to evaluate the direction of effect between lifetime smoking behavior and DNA methylation at the identified CpG sites. For lifetime smoking behavior, we obtained summary statistics for 126 independent SNPs identified in a GWAS of comprehensive smoking index,[29] with $p < 5 \times 10^{-8}$. We looked up these SNPs in a GWAS of DNA methylation at the CpG sites of interest, as described above. We then conducted MR to appraise the causal effect of lifetime smoking behavior on DNA methylation. We also looked up mQTLs that proxied for DNA methylation at the CpG sites of interest in the summary data from the GWAS of lifetime smoking behavior and conducted another two-sample MR analysis to appraise the causal effect of DNA methylation on lifetime smoking behavior.

Negative Control

We also assessed the association between the mQTL and DNA methylation at the CpG sites of interest by using data from the childhood time point of ALSPAC and exact linear regression as described above. This can be viewed as a negative-control analysis assessing the specificity of the mQTL effect on DNA methylation because the association should not be present in this group of non-smoking individuals if it is driven by smoking behavior.

Mediation Analysis

For those CpG sites where there was consistent evidence that methylation had a causal effect on lung function and where lifetime smoking was also causally implicated, we used a two-step MR approach[12] to investigate mediation. Prior to this, we performed an MR analysis to estimate the total causal effect of lifetime smoking behavior on lung function by looking up the SNPs associated with lifetime smoking behavior in the GWAS summary data for FEV_1.

For those CpGs where there was evidence that smoking influenced DNA methylation, which in turn influenced lung function, we used the "product of coefficients" method[35] to obtain an estimate for the indirect effect of smoking on lung function via DNA methylation. For those CpGs where there was evidence that, conversely, DNA methylation influenced smoking, which in turn influenced lung function, we used the "product of coefficients" method to obtain an estimate for the indirect effect of DNA methylation on lung function via smoking. This approach is outlined in Figure 1. Standard errors for the indirect effect were derived by using the delta method.

Another MR approach that can be used for assesing mediation is multivariable MR (MVMR).[36,37] This approach can help researchers to determine the direct effect of an exposure on an outcome, which can be subtracted from the total effect to obtain an estimate for the indirect effect ("difference in coefficients method").[35] We used MVMR to estimate the direct effects of lifetime smoking and the identified CpG sites on lung function by including the genetic proxies for smoking and each CpG site in turn in the multivariable models. SEs for the indirect effect were derived with the delta method.

Evaluating Horizontal Pleiotropy

Although various sensitivity analyses for investigating horizontal pleiotropy in MR analysis exist,[15] these approaches typically rely upon the existence of multiple genetic proxies associated with

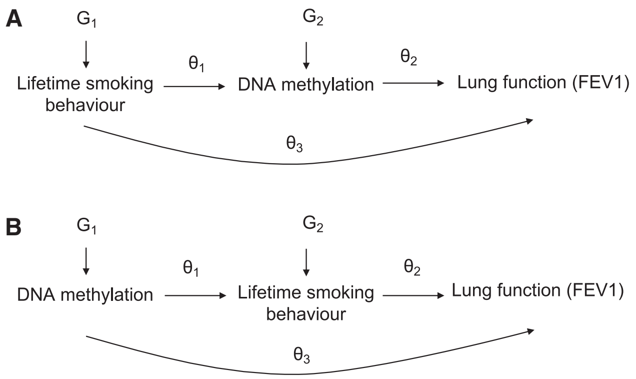

Figure 1. Outline of the Steps of the Mediation Analysis
(A) Assessment of the mediating role of DNA methylation in the effect of smoking behavior on lung function (FEV_1).
(B) Assessment of the mediating role of smoking behavior in the effect of DNA methylation on lung function (FEV_1). θ_1 = step 1; θ_2 = step 2; indirect effect = $\theta_1 \times \theta_2$ (product of coefficients); direct effect = θ_3; total causal effect = $\theta_3 + \theta_1$ x θ_2.

each exposure. Because only a small number of independent mQTLs are usually associated with individual CpG sites, it is often difficult to evaluate horizontal pleiotropy. To overcome this, we used an approach whereby we could examine multiple mQTLs in linkage disequilibrium ($r^2 < 0.8$) as instruments for a given CpG site and incorporated the correlation of the mQTLs as weights in a weighted generalized linear regression.[38] This was performed with summary statistics from Matrix eQTL,[10] as well as the "LDlink" and "MendelianRandomization" packages in R (version 3.5.1). Further to this, we assessed overall horizontal pleiotropy by (1) quantifying the heterogeneity of the genetic variants based on the Q statistic by using modified weights for the IVW approach,[39] as well as the MR-PRESSO global test,[40] and (2) testing the intercept in the MR-Egger test.[41] To account for horizontal pleiotropy, we performed two additional MR analyses that make different assumptions about this: (1) MR Egger regression[41] and (2) the weighted median approach.[42] The following R packages were used for these analyses: "Mendelian Randomization," "RadialMR," and "MR-PRESSO."

Multiple-Trait Colocalization Analysis

For those CpG sites where there was evidence of a causal effect on lung function, we applied ("moloc")[18] to investigate whether the variant responsible for influencing methylation at each CpG site was the same variant influencing changes to both nearby gene expression and lung function.[17,43] We applied "moloc" by using data derived from three different sources: mQTL data from the middle-age time point (mean age 47.5 years) in ARIES, GWAS summary data for FEV_1 from the UK Biobank,[20] and expression quantitative-trait loci (eQTL) data derived from whole blood from the eQTLGen Consortium (n = 31,684).[44] We ran "moloc" multiple times to investigate colocalization with the expression of all genes within 1 Mb of the CpG site of interest. Analyses were only undertaken if there were at least 50 variants (minor-allele frequency [MAF] ≥ 5%) in common between all three datasets. As recommended by the developers of "moloc", a posterior probability of association (PPA) of 80% or higher was considered evidence of colocalization. This approach therefore suggests that loci with evidence of genetic colocalization harbor a single causal variant that is responsible for variation in DNA methylation, gene expression, and lung function. When there was evidence at the same locus with multiple genes, we reported the association with the highest PPA. All analyses were undertaken with R (version 3.5.1).

Results

Analysis Pipeline

A summary of the analysis pipeline used to investigate the causal effect of DNA methylation on lung function is shown in Figure 2.

Discovery Analysis

We first identified mQTLs that could serve as proxies for 2,622 smoking-related CpG sites identified in a large epigenome-wide association study (EWAS) meta-analysis conducted by the CHARGE Consortium (Table S1).[3] For this, we used a catalog of SNPs associated with CpG sites in the ARIES study[10] to identify conditionally independent mQTLs (from genome-wide complex-trait analysis) from the middle-age time point (mean age 47.5 years, n = 846).[10] We were able to proxy 474 unique CpG sites associated with smoking ($p < 1 \times 10^{-7}$) by using at least one mQTL (96% in *cis*, 4% in *trans*). 406 of the 474 CpGs (86%) were proxied by a single SNP, of which 16 (4%) were in *trans* (Table S2). Of these, 415 were present in a FEV_1 GWAS (n = 321,047) conducted as part of the UK Biobank study. The minimum r^2 for an mQTL was 2.9%, and the minimum F-statistic was 10.29, and the mean r^2 was 9.9% and mean F-statistic was 109.4, thus indicating adequate strength of the genetic variants for MR analysis (Table S2).

To assess the causal effect of DNA methylation at smoking-related CpG sites on lung function, we looked up the identified mQTLs in the lung-function GWAS summary data from the UK Biobank and conducted two-sample MR. We observed 18 CpG-FEV_1 effect estimates that survived multiple-testing correction (Bonferroni $p < 1.2 \times 10^{-4}$) (Table 1, Table S3), and we found evidence for more causal effects than would be expected on the basis of chance (Figure S1).

Given previous findings of a mediating role of *AHRR* (cg05575921) methylation in the relationship between smoking and lung function, we specifically tested the causal effect of methylation at cg05575921 on FEV_1 in an MR framework. Because no mQTLs were found to be robustly associated with this CpG site in the middle age time point of ARIES, we identified two mQTLs from the ARIES childhood time point and carried these forward to the MR analysis (Table S4). This revealed no strong evidence for a causal effect of *AHRR* (cg05575921) methylation on FEV_1 (Table S5).

Replication Analysis

We attempted to replicate effect estimates for the top 18 CpG sites identified in the UK Biobank by using data from the SpiroMeta GWAS meta-analysis of FEV_1 (n = 79,055) (Figure 3). Three CpGs (cg21201401 [*LIME1/ZGPAT*], cg19758448 [*PGAP3*], and cg12616487

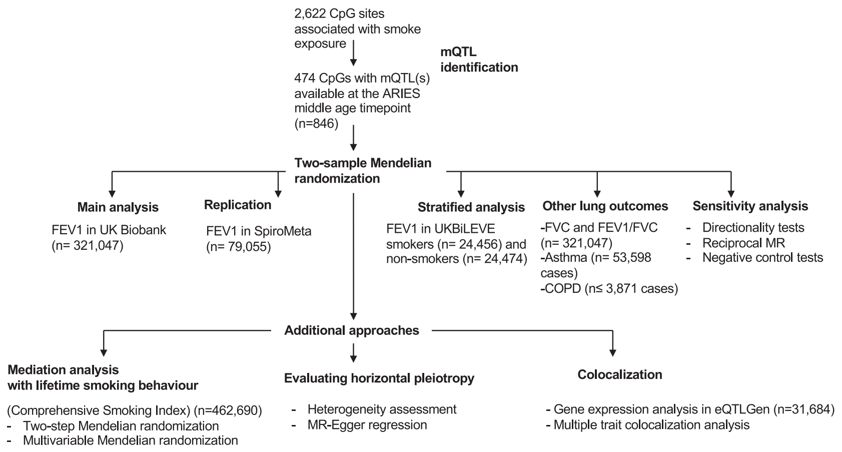

Figure 2. Flowchart of the Analysis Pipeline, Outlining the Different Analyses Performed at Each Stage of the Study
Cohorts and sample sizes used for each analysis are detailed in the flowchart.

[*EML3/AHNAK*]) were replicated beyond a stringent Bonferroni threshold ($p < 0.0028$) (Table S6), and there was consistency (83%) in the direction of effect at 15 of the CpG sites.

Stratified Analysis

The sample used in the discovery analysis included current, former, and never smokers in the UK Biobank, and so we performed a stratified analysis by using never- and heavy-smoking subsets of the UK Biobank study in the UK BiLEVE dataset. This stratified analysis had less statistical power than the discovery analysis as a result of a 10-fold drop in sample size (smokers, n = 24,457; non-smokers, n = 24,474). The results from the stratified analysis are compared to the discovery analysis in Figure S2, and Table S7 shows the results for the top CpGs. Effect estimates were generally similar between the mixed, smoking-only, and non-smoking-only groups. For some sites (cg09099830, cg09206294, cg15951188, cg24033122, and cg10672416), an effect was present in smokers but not in non-smokers, whereas at others (cg10255761, cg0632664, cg21201401, and cg04337534), there was a larger effect in non-smokers than in smokers.

Causal Effects of DNA Methylation on Other Lung-Function-Related Traits

Of the CpG sites where DNA methylation was identified as having a putative causal role on FEV_1, there was similar evidence for a causal effect of DNA methylation on FVC at 15 CpG sites beyond a stringent Bonferroni threshold ($p < 0.0028$) and on the FEV_1/FVC ratio at eight CpG sites (Figure S3, Table S8). Evidence for a causal effect on lung diseases (i.e., asthma and COPD) at these sites was not as strong, although this analysis was less well powered (Figure S4, Table S8). Nonetheless, four CpG sites (cg09447622, cg10672416, cg19758448, and cg21201401) surpassed the Bonferroni threshold in relation to asthma, and the effects observed for both asthma and COPD were typically in the opposite direction of those for the lung-function measures, as expected.

DNA Methylation and Lung Function: Direction of Causality

We performed directionality tests by using the MR Steiger method[34] to provide evidence that the causal pathway was in the direction from DNA methylation to FEV_1, rather than vice versa. This was suggested to be the case for all CpG sites in the main analysis (Table S9) because the mQTLs explained substantially more variation in DNA methylation (between 3.3% for cg09447622 and 31.3% for cg24033122) than in FEV_1 ($r^2 < 0.04\%$). When testing the impact of FEV_1 on DNA methylation, we used 175 out of 221 SNPs identified in the UK Biobank GWAS[20] as genetic proxies and found little evidence to suggest that lung function had a causal effect on DNA methylation at any of the 18 CpG sites (Table S10).

Smoking Behavior and DNA Methylation: Direction of Causality

We also evaluated the direction of causality between lifetime smoking behavior and DNA methylation at the identified CpG sites by using 119 out of 126 SNPs identified from a GWAS of a comprehensive smoking-index metric[29] as genetic proxies. There was limited evidence that lifetime smoking behavior had a causal effect on DNA methylation at the 18 CpG sites of interest, and the effect estimate from MR analysis was consistent with the original smoking EWAS at only 12 of the 18 CpG sites in terms of the direction of methylation (Table S11). This finding is in contrast to MR analysis for the majority of the smoking-related CpG sites, where the MR estimates were more in line with those from the smoking EWAS (Figure S5).

Conversely, there was evidence for a causal effect of DNA methylation on lifetime smoking at several of the CpG sites when we performed the reciprocal MR analysis (Table S12). We also performed directionality tests by using the MR Steiger method, which provided evidence that the causal pathway was in the direction from DNA methylation to smoking, rather than vice versa (Table S13).

Negative Control

Given the differences in sample sizes between the DNA methylation and lifetime smoking datasets that may bias the directionality tests,[34] we also carried out further analysis using mQTL data from the childhood time point (mean age 7.5 years, n = 885) as a negative control. We showed that the mQTLs were strongly associated with

Table 1. Results of Two-Sample MR Analysis of the Effects on Lung Function (FEV_1) of DNA Methylation at Smoking-Related CpG Sites.

CpG	Chromosome	Position	Nearest Gene(s)	Method	N SNPs	b	SE	p Value
cg12616487	11	62379063	*EML3/AHNAK*	Wald ratio	1	−0.101	0.010	3.34×10^{-24}
cg09447622	6	35108605	*TCP11*	Wald ratio	1	0.063	0.009	4.77×10^{-12}
cg21201401	20	62367884	*LIME1/ZGPAT*	Wald ratio	1	0.076	0.013	1.54×10^{-9}
cg19758448	17	37828296	*PGAP3*	Wald ratio	1	0.029	0.005	6.98×10^{-9}
cg06382664	11	73098877	*RELT*	Wald ratio	1	−0.045	0.008	1.16×10^{-8}
cg24033122	16	30485383	*ITGAL*	Wald ratio	1	0.019	0.004	2.31×10^{-7}
cg09099830	16	30485485	*ITGAL*	Wald ratio	1	0.042	0.008	2.57×10^{-7}
cg21356710	2	24234017	*MFSD2B*	Wald ratio	1	0.030	0.006	5.19×10^{-7}
cg10672416	12	123718706	*C12orf65*	Wald ratio	1	0.043	0.009	8.97×10^{-7}
cg15059804	1	33766318	*ZNF362*	Wald ratio	1	−0.023	0.005	2.02×10^{-6}
cg10255761	3	49210029	*KLHDC8B*	Wald ratio	1	0.042	0.009	2.48×10^{-6}
cg23771366	11	86510998	*PRSS23*	Wald ratio	1	0.039	0.008	2.59×10^{-6}
cg09206294	15	42072687	*MAPKBP1*	Wald ratio	1	−0.049	0.011	2.95×10^{-6}
cg15233611	12	122244660	*SETD1B*	Wald ratio	1	0.051	0.011	3.09×10^{-6}
cg19717773	7	2847554	*GNA12*	Inverse-variance weighted	2	−0.032	0.007	6.94×10^{-6}
cg04337534	11	65816809	*GAL3ST3*	Wald ratio	1	0.052	0.012	1.16×10^{-5}
cg15951188	17	7832680	*KCNAB3*	Wald ratio	1	−0.023	0.005	3.27×10^{-5}
cg11660018	11	86510915	*PRSS23*	Wald ratio	1	0.036	0.009	5.54×10^{-5}

Two-sample MR analysis involving SNP-methylation estimates from ARIES (sample 1, Table S2) and SNP-FEV_1 estimates from the UK Biobank (sample 2). The effect size (b), standard error (SE), and p value for each CpG reaching significance after Bonferroni correction is reported, along with the chromosome and position of the CpG, the nearest gene(s), the MR method used for analyzing the effect on lung function, and the number of SNPs used.

DNA methylation in ARIES at the childhood time point (i.e., in non-smoking individuals) and thereby ruled out the possibility that the mQTLs were having their primary effect via smoking (Table S14).

Mediation Analysis

Given the limited evidence suggesting that smoking has a causal effect on DNA methylation at the 18 CpG sites of interest, we conducted mediation analysis to investigate the mediating pathway from DNA methylation to lung function via lifetime smoking behavior at the seven CpG sites where there was evidence for a causal effect of DNA methylation on lung function, as well as smoking behavior beyond a Bonferroni threshold ($p < 0.0028$) (Table S12).

We accomplished this by performing two-step MR analysis[12] (Figure 1) and using the "product of coefficients" method[35] to estimate the indirect effect of DNA methylation on lung function via lifetime smoking. For all seven CpG sites, there was evidence of an indirect effect of DNA methylation on lung function via lifetime smoking ($p \leq 0.006$). Between 7.85% and 19.33% of the total effect was found to be mediated by each of the CpG sites (Table S15). This indirect effect was replicated for five CpG sites when we used FEV_1 GWAS summary data from SpiroMeta ($p \leq 0.008$) (Table S16).

We also estimated the direct effect of methylation on lung function by using an MVMR approach,[36,37] and we used the "difference of coefficients" method[35] to determine the indirect effect of DNA methylation on lung function via lifetime smoking (Tables S17 and S18). Although independent strength of the genetic variants for lifetime smoking and the 18 CpG sites was deemed to be strong (Q-statistics $\geq$ 64.5), the indirect effect was estimated with lower precision than the two-step MR analysis. In addition, there was some evidence for heterogeneity in the causal-effect estimates from the MVMR, which could indicate the presence of invalid genetic variants (e.g., as a result of horizontal pleiotropy)[37] (Table S18). Nonetheless, there was supportive evidence for an indirect effect of methylation at two sites (cg10255761 and cg15951188) on FEV_1 via smoking in MVMR (Tables S17 and S18).

Evaluating Horizontal Pleiotropy

For the 18 CpG sites of interest, we assessed the robustness of the causal effects to horizontal pleiotropy by using multiple mQTLs in linkage disequilibrium ($r^2 < 0.8$) as proxies for each CpG site, and we incorporated the correlation of the mQTLs as weights in a weighted generalized linear-regression analysis.[38] Given the presence of more than one mQTL, we were able to perform IVW and compared the results with those obtained from the main analysis involving only independent mQTLs (Figure S6, Table S19). Evidence for horizontal pleiotropy was evident for two of the CpG sites (cg10255761 and cg21201401) on

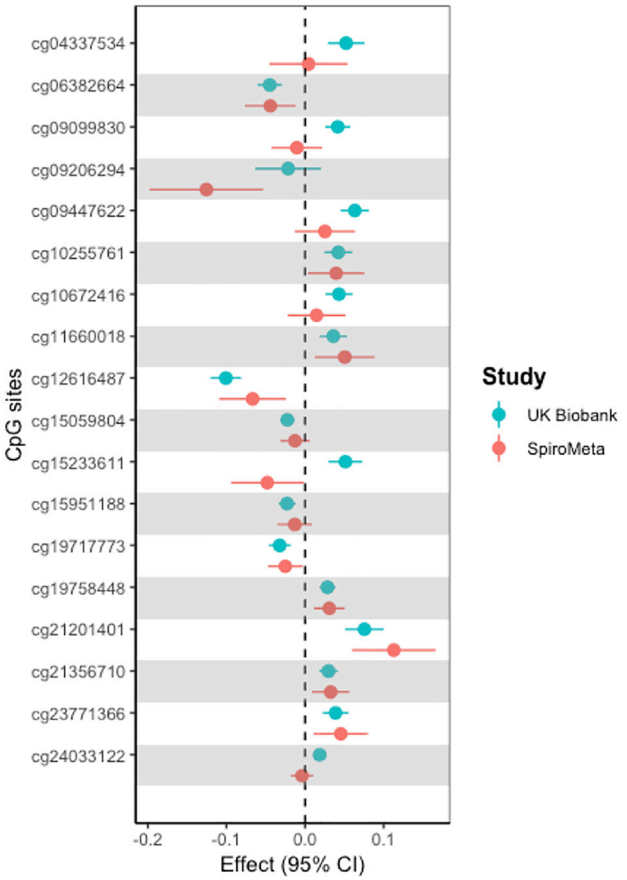

Figure 3. Results of MR Analysis of the Effect of Smoking-Associated DNA Methylation on Lung Function (FEV_1) in the UK Biobank (Discovery) and SpiroMeta (Replication) Datasets
Effect sizes and 95% confidence intervals (CI) of the 18 significant CpG sites from the discovery analysis are shown in blue, and the effect sizes and CI of the same CpG sites in the replication analysis in SpiroMeta are shown in red.

the basis of heterogeneity assessment and for five CpGs (cg10672416, cg19758448, cg21201401, cg23771366, and cg24033122) on the basis of the MR Egger intercept value at a Bonferroni threshold of $p < 0.0028$ (Figure S7, Table S20). After we accounted for horizontal pleiotropy, effects at cg10672416, cg23771366, and cg24033122 were attenuated in the MR Egger regression, whereas at cg21201401, evidence suggested a causal effect of DNA methylation on FEV_1 in the opposite direction to that estimated in the other analyses (Figure S8). Effect estimates based on the weighted median approach were largely consistent with those from IVW.

Multiple-Trait Colocalization Analysis

For those CpG sites where there was evidence of a causal effect on lung function, we applied a genetic colocalization approach to determine whether the variant responsible for influencing methylation at each CpG site was the same variant influencing changes in lung function. Furthermore, it is likely that any true association between a CpG site and lung function is mediated by changes to the expression of nearby genes. To assess this, we applied "moloc"[18] to investigate whether the variant responsible for influencing methylation at each CpG site was the same variant influencing changes to both nearby gene expression and lung function.[17,43]

There was strong evidence (based on PPA ≥ 80%) at five CpG sites that variation in DNA methylation, gene expression, and FEV_1 were all attributed to the same underlying genetic variant. This included associations at cg21201401 (with *ZGPAT* expression [PPA = 84.2%]) and cg12616487 (with *AHNAK* expression [PPA = 88.9%]), which were two of the CpGs where the effects on FEV_1 were most strongly replicated in SpiroMeta. This suggests that the relationship between DNA methylation at these smoking-associated CpG sites and lung function might also involve the transcription of nearby genes, and such transcription is a mechanism of effect consistent with causality. There was also strong evidence of colocalization at four further CpG sites, although only between DNA methylation and FEV_1 (but not nearby gene expression). Colocalization results are shown in Table S19. We note, however, that plotting genetic effects at each of these loci highlighted that many of them were in regions of high linkage disequilibrium (Figure S9).

Discussion

We investigated CpG sites previously associated with smoking for their potential causal impact on lung function by using a two-step MR framework. A discovery MR analysis involving mQTLs associated with 474 smoking-associated CpGs identified 18 CpGs with a possible causal effect on lung function in the UK Biobank. These sites were annotated to genes involved in diverse biological pathways, including neurological development (*AHNAK*, *PGAP3*), lymphocytic function (*ITGAL*), apoptosis (*RELT*), tumor suppression (*ZGPAT*), and endothelial-to-mesenchymal transition (*PRSS23*). Genetic variation in *ZNF362* has also been recently implicated in relation to risk-taking propensity.[45] Replication in SpiroMeta provided supportive evidence for a causal effect of methylation on FEV_1 at three CpG sites, although the sample size of this replication analysis was much smaller than that performed with UK Biobank data (79,055 versus 321,047), and there was consistency in the direction of effect at 83% of the CpG sites. A further analysis using the UK BiLEVE dataset stratified by smoking status highlighted heterogeneity in effects among heavy smokers compared with non-smokers at some of the sites. 15 of the CpG sites identified in relation to FEV_1 also showed evidence for a causal effect on FVC, and eight showed an effect on the FEV_1/FVC ratio. There was also suggestive evidence for causality on lung diseases (i.e., asthma and COPD).

We found little evidence to suggest that lung function in turn influenced DNA methylation at the 18 CpG sites. Interestingly, MR analyses also provided limited evidence

that smoking had a causal effect on DNA methylation at these smoking-related sites. Instead, we observed that at several of the CpG sites DNA methylation had a causal effect on smoking. We conducted mediation analysis by using both two-step and multivariable MR to estimate the extent to which smoking mediates the association between DNA methylation and lung function at these sites. In two-step MR, we found evidence of mediation for seven CpG sites when we used FEV_1 GWAS summary data from the UK Biobank and for five CpG sites when we used SpiroMeta. Indirect effects were estimated with less precision in the MVMR approach. We also performed additional MR analyses to investigate horizontal pleiotropy, and we integrated evidence from gene expression in "moloc" to provide further evidence for causality.

Comparison with Other Studies

We searched both the EWAS Catalog and the EWAS Atlas[46] to assess whether any of the 18 CpG sites had been previously identified in other EWASs of lung function or COPD. The CpG sites cg15059804 (*ZNF362*) and cg11660018 (*PRSS23*) were found to be associated with asthma in an EWAS conducted in lung cells;[47] cg11660018 (*PRSS23*) and cg23771366 (*PRSS23*) were suggested to have a causal effect on lung function in another EWAS conducted in blood; this study was followed up by a two-sample MR analysis.[7] The direction of causal effect for these two CpGs in this MR analysis was consistent with our results. cg21201401 (*LIME1/ZGPAT*) was found to be inversely associated with COPD in an EWAS conducted in lung tissue (114 subjects with COPD and 46 controls who were all former smokers).[48] This effect is consistent with our observation of a causal effect on increased FEV_1.

As mentioned in the Introduction, one previous study indicated that hypomethylation at cg05575921 (*AHRR*) might mediate the association between smoking and lung function.[7] However, we found in MR analysis that there was no strong evidence for a causal effect of *AHRR* methylation on FEV_1, indicating that it is unlikely to be mediating the effect of smoking on lung function. Similar conflicting findings have been observed between conventional mediation approaches and MR analysis aimed at determining epigenetic mediation in the context of smoking and lung cancer[49,50] and of prenatal famine and later-life metabolic profile.[51,52] Traditional mediation approaches are more susceptible to measurement error and potential reverse causation than MR,[53] meaning the proportion of the mediated effect reported by these studies is likely to be overestimated. However, several limitations of MR analysis have also been raised previously and might explain discrepancies, including tissue specificity, pleiotropy, and low power, in these results.[54] These limitations are discussed in turn below.

Limitations

Sample Considerations

A possible explanation for why this MR analysis did not detect a causal effect of smoking on DNA methylation is low power resulting from the small sample size for the DNA methylation sample (n = 846). Three of the 18 sites identified as having a causal effect on lung function in our analysis were also previously implicated in an EWAS of maternal smoking in pregnancy,[55] although the direction of effect was not always consistent with our results. These were cg12616487 (*EML3/AHNAK*), cg23771366 (*PRSS23*), and cg21201401 (*LIME1/ZGPAT*). Because DNA methylation is unlikely to directly influence maternal smoking in this instance, this indicates that smoke exposure (whether this be through one's own smoking or smoke exposure in-utero) might have a causal effect on DNA methylation but that this effect might have been undetected in our MR analysis. Furthermore, the intergenerational effect that maternal smoking had at these CpG sites might have biased the negative-control analysis in that the mQTL effect seen in childhood could have been confounded by parental smoking and inherited mQTLs.

Furthermore, although both the GWASs for lifetime smoking and lung function were conducted in samples that included both males and females, the mQTL effects used in the main analysis were obtained in females only in ARIES. Nonetheless, we have shown consistency in the mQTL effects in a mixed sample of males and females from the ARIES childhood time point.

An additional sample consideration relates to the use of both the UK Biobank and the UKBiLEVE subset, both of which represent selected groups that could bias effect estimates in the MR analysis.[56] Nonetheless, we have also performed independent replication by using data from 22 studies from the SpiroMeta Consortium, and these provided confirmatory causal estimates at the majority of the identified CpG sites.

Horizontal Pleiotropy

We observed heterogeneity of causal effects for some of the CpG sites between smokers and non-smokers. For example, at cg10255761 (*KLHDC8B*) and cg21201401 (*LIME1/ZGPAT*), although DNA methylation was shown to have a causal effect on smoking and lung function in the mediation analysis, there was also evidence for a causal effect on lung function among non-smokers. Caution over these results is warranted, since this stratified analysis effectively conditions on a collider (i.e., smoking status) that might induce bias.[57] However, another potential explanation for these findings is the horizontal pleiotropy in the MR analysis. We performed additional MR analyses to detect and correct for this bias, and we demonstrated that at some CpG sites, including cg21201401 (*LIME1/ZGPAT*), there was evidence to suggest horizontal pleiotropy.

Multiple-Trait Colocalization

We also performed a colocalization analysis on our top hits to investigate the relationship between methylation of these sites, expression of nearby genes, and variation in lung function. If all three of these traits were to share a common causal variant, it would suggest that associations are more likely to be due to an underlying causal

relationship as opposed to genetic confounding (i.e., high linkage disequilibrium between an mQTL and a variant that influences lung function).

Our colocalization analysis revealed that genetic variation associated with DNA methylation colocalizes with both variation in lung function and gene expression at several sites. For example, methylation at cg21201401 was shown to colocalize with *ZGPAT* expression and lung function, and methylation at cg12616487 was shown to colocalize with *AHNAK* expression and lung function. Although findings related to cg21201401 and *ZGPAT* expression should be interpreted with caution, given the presence of horizontal pleiotropy in the MR analysis, *AHNAK* is a strong candidate for being responsible for the association of this locus with variation in lung function. In particular, *AHNAK* is responsible for a neuroblast differentiation-associated protein that has previously been reported to confer risk of COPD as the result of missense variants in its coding region.[58] However, it should be noted that several of the mQTLs investigated in the colocalization analysis were in regions of high linkage disequilibrium. As such, although the findings might be useful in prioritizingloci where epigenetic factors putatively influence variation in lung function, functional studies will need to robustly demonstrate this. Furthermore, evidence for a causal effect of DNA methylation at cg12616487 (*EML3/AHNAK*) was replicated in an MR analysis with data from the SpiroMeta Consortium. This further supports evidence indicating that it represents a promising candidate for being a potential molecular mediator along the causal pathway from smoking to variation in lung function

We detected evidence of colocalization between DNA methylation and lung function at various CpG sites, but gene expression did not also colocalize with these. For example, the functional gene that might be responsible for the association at cg21356710 could be *UBXN2A* because, although it is not the closest gene to the CpG site, it has been previously implicated in nicotine metabolism.[59] However, strong evidence from future research would need to support this.

Tissue Specificity

A recent study that investigated the colocalization of mQTLs with genetic risk variants for COPD identified several lung-tissue mQTLs that might be involved in COPD pathogenesis.[60] These findings did not overlap with the findings of this study, perhaps because of differences in tissue type. However, because some of the CpG sites that were causally implicated in our MR analysis might be exerting their effect on lung function via smoking behavior, lung tissue might not always be the most relevant for appraising causal effects. Future work should evaluate and integrate mQTL and eQTL effects from multiple tissues to elucidate causal effects in the most biologically relevant tissues. For example, one could use lung-derived tissue to perform an investigation similar to that undertaken in our study in order to further evaluate the molecular mechanisms that influence lung function.

Measurement Imprecision

One of the main limitations of mediation analysis is the assumption of no measurement error. MR attempts to overcome this limitation with the use of genetic variants, which are typically measured with high accuracy. However, differential measurement precision of the phenotypes being investigated in an MR approach can lead to spurious findings in certain instances.

One explanation for the finding that DNA methylation has a causal effect on smoking at several of the CpG sites is that the SNPs used to proxy for DNA methylation have their primary effect through smoking. We assessed this by using the Steiger test, which indicated that this alternative explanation was not likely for those CpG sites where DNA methylation had a causal effect on smoking. However, this test is liable to return inaccurate causal directions if there are large differences in sample size between the two samples or if the phenotypes have differences in measurement precision,[34] which is likely to be the case in this context. To assess this further, we compared the magnitude of the mQTL effects in a nonsmoking subset of ARIES (children at age 7 years) and found similar effects.

Strengths

Despite these limitations, this study has several strengths, which include the systematic evaluation of the causal effect of a large number of smoking-related CpG sites on lung function; the replication of findings in different smoking strata and in an independent dataset; the integration of several large-scale datasets in the evaluation of the causal relationship between smoking, DNA methylation, and lung function; the application of a formal two-step MR approach in the evaluation of mediation; and the use of a colocalization approach that integrated gene expression data.

Conclusions

Using an MR approach, we identified several CpG sites where DNA methylation might have a causal effect on lung function, as assessed by FEV_1. At some sites, there was evidence to suggest that DNA methylation influenced smoking, which in turn influenced lung function, rather than that smoking influenced DNA methylation, which then influenced lung function, as in the originally proposed mechanism. The findings presented here highlight potential therapeutic targets for improving lung function and possibly smoking cessation, although further studies with larger-scale and tissue-specific DNA methylation and expression data will need to confirm these results.

Supplemental Data

Supplemental Data can be found online at https://doi.org/10.1016/j.ajhg.2020.01.015.

Acknowledgments

We thank the SpiroMeta Consortium for contributing summary statistics to this work. We would also like to thank Alice Carter, Dipender Gill, and Eleanor Sanderson for useful discussions regarding the mediation analysis. This study was made possible with the financial support of Jonathan de Pass and Georgina de Pass. This work was supported by the Integrative Epidemiology Unit, which receives funding from the UK Medical Research Council and the University of Bristol (MC_UU_00011/1, MC_UU_00011/3, MC_UU_00011/4, MC_UU_00011/5, and MC_UU_00011/7). This work was also supported by CRUK (grant number C18281/A19169) and the ESRC (grant number ES/N000498/1). T.B. and R.K.L. are supported by Wellcome Trust PhD studentships (203746 and 215193/Z18/Z). T.G.R. is a UKRI Innovation Research Fellow (MR/S003886/1). R.C.R. is a de Pass Vice Chancellor Research Fellow at the University of Bristol. A.L.G. is funded by internal fellowships at the University of Leicester for the Wellcome Trust Institutional Strategic Support Fund (WT204801/Z/16/Z) and the BHF Accelorator Award (AA/18/3/34220). M.D.T. is supported by a Wellcome Trust Investigator Award (WT202849/Z/16/Z). The research was partially supported by the NIHR Biomedical Research Centre at the University Hospitals Bristol NHS Foundation Trust and the NIHR Leicester Biomedical Research Centre: the views expressed in this publication are those of the authors and not necessarily those of the NHS, the NIHR, or the Department of Health.

Declaration of Interests

M.D.T. has received grant support from GSK and Orion Pharma. All other authors declare no competing interests.

Received: August 20, 2019
Accepted: January 21, 2020
Published: February 20, 2020

Web Resources

ALSPAC data dictionary, http://www.bristol.ac.uk/alspac/researchers/our-data/
ALSPAC, http://www.bristol.ac.uk/alspac
ARIES mQTL database, http://mqtldb.org
EWAS Atlas, https://bigd.big.ac.cn/ewas
EWAS Catalog, http://www.ewascatalog.org/
UK Biobank study protocol, http://www.ukbiobank.ac.uk/wp-content/uploads/2011/11/UK-Biobank-Protocol.pdf

References

1. Lange, P., Celli, B., Agustí, A., Boje Jensen, G., Divo, M., Faner, R., Guerra, S., Marott, J.L., Martinez, F.D., Martinez-Camblor, P., et al. (2015). Lung-Function Trajectories Leading to Chronic Obstructive Pulmonary Disease. N. Engl. J. Med. *373*, 111–122.
2. Anthonisen, N.R., Connett, J.E., Kiley, J.P., Altose, M.D., Bailey, W.C., Buist, A.S., Conway, W.A., Jr., Enright, P.L., Kanner, R.E., O'Hara, P., et al. (1994). Effects of smoking intervention and the use of an inhaled anticholinergic bronchodilator on the rate of decline of FEV1. The Lung Health Study. JAMA *272*, 1497–1505.
3. Joehanes, R., Just, A.C., Marioni, R.E., Pilling, L.C., Reynolds, L.M., Mandaviya, P.R., Guan, W., Xu, T., Elks, C.E., Aslibekyan, S., et al. (2016). Epigenetic Signatures of Cigarette Smoking. Circ Cardiovasc Genet *9*, 436–447.
4. Bojesen, S.E., Timpson, N., Relton, C., Davey Smith, G., and Nordestgaard, B.G. (2017). *AHRR* (cg05575921) hypomethylation marks smoking behaviour, morbidity and mortality. Thorax *72*, 646–653.
5. Barfield, R., Shen, J., Just, A.C., Vokonas, P.S., Schwartz, J., Baccarelli, A.A., VanderWeele, T.J., and Lin, X. (2017). Testing for the indirect effect under the null for genome-wide mediation analyses. Genet. Epidemiol. *41*, 824–833.
6. London, S.J. (2019). Methylation, smoking, and reduced lung function. Eur. Respir. J. *54*, 1900920.
7. de Vries, M., van der Plaat, D.A., Nedeljkovic, I., Verkaik-Schakel, R.N., Kooistra, W., Amin, N., van Duijn, C.M., Brandsma, C.A., van Diemen, C.C., Vonk, J.M., and Marike Boezen, H. (2018). From blood to lung tissue: effect of cigarette smoke on DNA methylation and lung function. Respir. Res. *19*, 212.
8. Imboden, M., Wielscher, M., Rezwan, F.I., Amaral, A.F.S., Schaffner, E., Jeong, A., Beckmeyer-Borowko, A., Harris, S.E., Starr, J.M., Deary, I.J., et al. (2019). Epigenome-wide association study of lung function level and its change. Eur. Respir. J. *54*, 1900457.
9. Smith, G.D., and Ebrahim, S. (2003). 'Mendelian randomization': can genetic epidemiology contribute to understanding environmental determinants of disease? Int. J. Epidemiol. *32*, 1–22.
10. Gaunt, T.R., Shihab, H.A., Hemani, G., Min, J.L., Woodward, G., Lyttleton, O., Zheng, J., Duggirala, A., McArdle, W.L., Ho, K., et al. (2016). Systematic identification of genetic influences on methylation across the human life course. Genome Biol. *17*, 61.
11. Davey Smith, G., and Hemani, G. (2014). Mendelian randomization: genetic anchors for causal inference in epidemiological studies. Hum. Mol. Genet. *23* (R1), R89–R98.
12. Relton, C.L., and Davey Smith, G. (2012). Two-step epigenetic Mendelian randomization: a strategy for establishing the causal role of epigenetic processes in pathways to disease. Int. J. Epidemiol. *41*, 161–176.
13. Pierce, B.L., and Burgess, S. (2013). Efficient design for Mendelian randomization studies: subsample and 2-sample instrumental variable estimators. Am. J. Epidemiol. *178*, 1177–1184.
14. Burgess, S., Daniel, R.M., Butterworth, A.S., Thompson, S.G.; and EPIC-InterAct Consortium (2015). Network Mendelian randomization: using genetic variants as instrumental variables to investigate mediation in causal pathways. Int. J. Epidemiol. *44*, 484–495.
15. Hemani, G., Zheng, J., Elsworth, B., Wade, K.H., Haberland, V., Baird, D., Laurin, C., Burgess, S., Bowden, J., Langdon, R., et al. (2018). The MR-Base platform supports systematic causal inference across the human phenome. eLife *7*, e34408.
16. Giambartolomei, C., Vukcevic, D., Schadt, E.E., Franke, L., Hingorani, A.D., Wallace, C., and Plagnol, V. (2014). Bayesian test for colocalisation between pairs of genetic association studies using summary statistics. PLoS Genet. *10*, e1004383.
17. Richardson, T.G., Haycock, P.C., Zheng, J., Timpson, N.J., Gaunt, T.R., Davey Smith, G., Relton, C.L., and Hemani, G. (2018). Systematic Mendelian randomization framework elucidates hundreds of CpG sites which may mediate the influence of genetic variants on disease. Hum. Mol. Genet. *27*, 3293–3304.
18. Giambartolomei, C., Zhenli Liu, J., Zhang, W., Hauberg, M., Shi, H., Boocock, J., Pickrell, J., Jaffe, A.E., Pasaniuc, B.,

Roussos, P.; and CommonMind Consortium (2018). A Bayesian framework for multiple trait colocalization from summary association statistics. Bioinformatics *34*, 2538–2545.

19. Richardson, T.G., Richmond, R.C., North, T.L., Hemani, G., Davey Smith, G., Sharp, G.C., and Relton, C.L. (2019). An integrative approach to detect epigenetic mechanisms that putatively mediate the influence of lifestyle exposures on disease susceptibility. Int. J. Epidemiol. *48*, 887–898.
20. Shrine, N., Guyatt, A.L., Erzurumluoglu, A.M., Jackson, V.E., Hobbs, B.D., Melbourne, C.A., Batini, C., Fawcett, K.A., Song, K., Sakornsakolpat, P., et al.; Understanding Society Scientific Group (2019). New genetic signals for lung function highlight pathways and chronic obstructive pulmonary disease associations across multiple ancestries. Nat. Genet. *51*, 481–493.
21. Boyd, A., Golding, J., Macleod, J., Lawlor, D.A., Fraser, A., Henderson, J., Molloy, L., Ness, A., Ring, S., and Davey Smith, G. (2013). Cohort Profile: the 'children of the 90s'–the index offspring of the Avon Longitudinal Study of Parents and Children. Int. J. Epidemiol. *42*, 111–127.
22. Fraser, A., Macdonald-Wallis, C., Tilling, K., Boyd, A., Golding, J., Davey Smith, G., Henderson, J., Macleod, J., Molloy, L., Ness, A., et al. (2013). Cohort Profile: the Avon Longitudinal Study of Parents and Children: ALSPAC mothers cohort. Int. J. Epidemiol. *42*, 97–110.
23. Relton, C.L., Gaunt, T., McArdle, W., Ho, K., Duggirala, A., Shihab, H., Woodward, G., Lyttleton, O., Evans, D.M., Reik, W., et al. (2015). Data Resource Profile: Accessible Resource for Integrated Epigenomic Studies (ARIES). Int. J. Epidemiol. *44*, 1181–1190.
24. Shabalin, A.A. (2012). Matrix eQTL: ultra fast eQTL analysis via large matrix operations. Bioinformatics *28*, 1353–1358.
25. Purcell, S., Neale, B., Todd-Brown, K., Thomas, L., Ferreira, M.A., Bender, D., Maller, J., Sklar, P., de Bakker, P.I., Daly, M.J., and Sham, P.C. (2007). PLINK: a tool set for whole-genome association and population-based linkage analyses. Am. J. Hum. Genet. *81*, 559–575.
26. Yang, J., Lee, S.H., Goddard, M.E., and Visscher, P.M. (2011). GCTA: a tool for genome-wide complex trait analysis. Am. J. Hum. Genet. *88*, 76–82.
27. Sudlow, C., Gallacher, J., Allen, N., Beral, V., Burton, P., Danesh, J., Downey, P., Elliott, P., Green, J., Landray, M., et al. (2015). UK biobank: an open access resource for identifying the causes of a wide range of complex diseases of middle and old age. PLoS Med. *12*, e1001779.
28. Bycroft, C., Freeman, C., Petkova, D., Band, G., Elliott, L.T., Sharp, K., Motyer, A., Vukcevic, D., Delaneau, O., O'Connell, J., et al. (2018). The UK Biobank resource with deep phenotyping and genomic data. Nature *562*, 203–209.
29. Wootton, R.E., Richmond, R.C., Stuijfzand, B.G., Lawn, R.B., Sallis, H.M., Taylor, G.M.J., Hemani, G., Jones, H.J., Zammit, S., Davey Smith, G., and Munafò, M.R. (2019). Evidence for causal effects of lifetime smoking on risk for depression and schizophrenia: a Mendelian randomisation study. Psychol. Med., 1–9.
30. Thomas, D.C., Lawlor, D.A., and Thompson, J.R. (2007). Re: Estimation of bias in nongenetic observational studies using "Mendelian triangulation" by Bautista et al. Ann. Epidemiol. *17*, 511–513.
31. Wain, L.V., Shrine, N., Artigas, M.S., Erzurumluoglu, A.M., Noyvert, B., Bossini-Castillo, L., Obeidat, M., Henry, A.P., Portelli, M.A., Hall, R.J., et al.; Understanding Society Scientific Group; and Geisinger-Regeneron DiscovEHR Collaboration (2017). Genome-wide association analyses for lung function and chronic obstructive pulmonary disease identify new loci and potential druggable targets. Nat. Genet. *49*, 416–425.
32. Wain, L.V., Shrine, N., Miller, S., Jackson, V.E., Ntalla, I., Soler Artigas, M., Billington, C.K., Kheirallah, A.K., Allen, R., Cook, J.P., et al.; UK Brain Expression Consortium (UKBEC); and OxGSK Consortium (2015). Novel insights into the genetics of smoking behaviour, lung function, and chronic obstructive pulmonary disease (UK BiLEVE): a genetic association study in UK Biobank. Lancet Respir. Med. *3*, 769–781.
33. Elsworth, B., Mitchell, R., Raistrick, C., Paternoster, L., Hemani, G., and Gaunt, T. (2019). MRC IEU UK Biobank GWAS pipeline version 2 (University of Bristol). https://research-information.bris.ac.uk/en/datasets/mrc-ieu-uk-biobank-gwas-pipeline-version-2(533d7172-cd33-4f9a-802e-0b612291b26a).html.
34. Hemani, G., Tilling, K., and Davey Smith, G. (2017). Orienting the causal relationship between imprecisely measured traits using GWAS summary data. PLoS Genet. *13*, e1007081.
35. VanderWeele, T.J. (2016). Mediation Analysis: A Practitioner's Guide. Annu. Rev. Public Health *37*, 17–32.
36. Burgess, S., and Thompson, S.G. (2015). Multivariable Mendelian randomization: the use of pleiotropic genetic variants to estimate causal effects. Am. J. Epidemiol. *181*, 251–260.
37. Sanderson, E., Davey Smith, G., Windmeijer, F., and Bowden, J. (2019). An examination of multivariable Mendelian randomization in the single-sample and two-sample summary data settings. Int. J. Epidemiol. *48*, 713–727.
38. Burgess, S., Dudbridge, F., and Thompson, S.G. (2016). Combining information on multiple instrumental variables in Mendelian randomization: comparison of allele score and summarized data methods. Stat. Med. *35*, 1880–1906.
39. Bowden, J., Del Greco M, F., Minelli, C., Zhao, Q., Lawlor, D.A., Sheehan, N.A., Thompson, J., and Davey Smith, G. (2019). Improving the accuracy of two-sample summary-data Mendelian randomization: moving beyond the NOME assumption. Int. J. Epidemiol. *48*, 728–742.
40. Verbanck, M., Chen, C.Y., Neale, B., and Do, R. (2018). Detection of widespread horizontal pleiotropy in causal relationships inferred from Mendelian randomization between complex traits and diseases. Nat. Genet. *50*, 693–698.
41. Bowden, J., Davey Smith, G., and Burgess, S. (2015). Mendelian randomization with invalid instruments: effect estimation and bias detection through Egger regression. Int. J. Epidemiol. *44*, 512–525.
42. Bowden, J., Davey Smith, G., Haycock, P.C., and Burgess, S. (2016). Consistent Estimation in Mendelian Randomization with Some Invalid Instruments Using a Weighted Median Estimator. Genet. Epidemiol. *40*, 304–314.
43. Richardson, T.G., Zheng, J., Davey Smith, G., Timpson, N.J., Gaunt, T.R., Relton, C.L., and Hemani, G. (2017). Mendelian Randomization Analysis Identifies CpG Sites as Putative Mediators for Genetic Influences on Cardiovascular Disease Risk. Am. J. Hum. Genet. *101*, 590–602.
44. Võsa, U., Claringbould, A., Westra, H.-J., Bonder, M.J., Deelen, P., Zeng, B., Kirsten, H., Saha, A., Kreuzhuber, R., Kasela, S., et al. (2018). Unraveling the polygenic architecture of complex traits using blood eQTL metaanalysis. bioRxiv. https://doi.org/10.1101/447367.
45. Clifton, E.A.D., Perry, J.R.B., Imamura, F., Lotta, L.A., Brage, S., Forouhi, N.G., Griffin, S.J., Wareham, N.J., Ong, K.K., and Day, F.R. (2018). Genome-wide association study for risk taking

propensity indicates shared pathways with body mass index. Commun. Biol. *1*, 36.

46. Li, M., Zou, D., Li, Z., Gao, R., Sang, J., Zhang, Y., Li, R., Xia, L., Zhang, T., Niu, G., et al. (2019). EWAS Atlas: a curated knowledgebase of epigenome-wide association studies. Nucleic Acids Res. *47* (D1), D983–D988.
47. Nicodemus-Johnson, J., Myers, R.A., Sakabe, N.J., Sobreira, D.R., Hogarth, D.K., Naureckas, E.T., Sperling, A.I., Solway, J., White, S.R., Nobrega, M.A., et al. (2016). DNA methylation in lung cells is associated with asthma endotypes and genetic risk. JCI Insight *1*, e90151.
48. Morrow, J.D., Cho, M.H., Hersh, C.P., Pinto-Plata, V., Celli, B., Marchetti, N., Criner, G., Bueno, R., Washko, G., Glass, K., et al. (2016). DNA methylation profiling in human lung tissue identifies genes associated with COPD. Epigenetics *11*, 730–739.
49. Fasanelli, F., Baglietto, L., Ponzi, E., Guida, F., Campanella, G., Johansson, M., Grankvist, K., Johansson, M., Assumma, M.B., Naccarati, A., et al. (2015). Hypomethylation of smoking-related genes is associated with future lung cancer in four prospective cohorts. Nat. Commun. *6*, 10192.
50. Battram, T., Richmond, R.C., Baglietto, L., Haycock, P.C., Perduca, V., Bojesen, S.E., Gaunt, T.R., Hemani, G., Guida, F., Carreras-Torres, R., et al. (2019). Appraising the causal relevance of DNA methylation for risk of lung cancer. Int. J. Epidemiol. *48*, 1493–1504.
51. Tobi, E.W., Slieker, R.C., Luijk, R., Dekkers, K.F., Stein, A.D., Xu, K.M., Slagboom, P.E., Van Zwet, E.W., Lumey, L.H., and Heijmans, B.T. (2018). DNA methylation as a mediator of the association between prenatal adversity and risk factors for metabolic disease in adulthood. Science Advances *4*, eaao4364.
52. Richmond, R.C., Timpson, N.J., and Sørensen, T.I.A. (2015). Exploring possible epigenetic mediation of early-life environmental exposures on adiposity and obesity development. Int. J. Epidemiol. *44*, 1191–1198.
53. Richmond, R.C., Relton, C.L., and Davey Smith, G. (2018). What evidence is required to suggest that DNA methylation mediates the association between prenatal famine exposure and adulthood disease? Sci. Adv., eaao4364. https://doi.org/10.1126/sciadv.aao4364.
54. Tobi, E.W., van Zwet, E.W., Lumey, L.H., and Heijmans, B.T. (2018). Why mediation analysis trumps Mendelian randomization in population epigenomics studies of the Dutch Famine. bioRxiv. https://doi.org/10.1101/362392.
55. Joubert, B.R., Felix, J.F., Yousefi, P., Bakulski, K.M., Just, A.C., Breton, C., Reese, S.E., Markunas, C.A., Richmond, R.C., Xu, C.J., et al. (2016). DNA Methylation in Newborns and Maternal Smoking in Pregnancy: Genome-wide Consortium Meta-analysis. Am. J. Hum. Genet. *98*, 680–696.
56. Munafò, M.R., Tilling, K., Taylor, A.E., Evans, D.M., and Davey Smith, G. (2018). Collider scope: when selection bias can substantially influence observed associations. Int. J. Epidemiol. *47*, 226–235.
57. Cole, S.R., Platt, R.W., Schisterman, E.F., Chu, H., Westreich, D., Richardson, D., and Poole, C. (2010). Illustrating bias due to conditioning on a collider. Int. J. Epidemiol. *39*, 417–420.
58. Nedeljkovic, I., Terzikhan, N., Vonk, J.M., van der Plaat, D.A., Lahousse, L., van Diemen, C.C., Hobbs, B.D., Qiao, D., Cho, M.H., Brusselle, G.G., et al. (2018). A Genome-Wide Linkage Study for Chronic Obstructive Pulmonary Disease in a Dutch Genetic Isolate Identifies Novel Rare Candidate Variants. Front. Genet. *9*, 133.
59. Teng, Y., Rezvani, K., and De Biasi, M. (2015). UBXN2A regulates nicotinic receptor degradation by modulating the E3 ligase activity of CHIP. Biochem. Pharmacol. *97*, 518–530.
60. Morrow, J.D., Glass, K., Cho, M.H., Hersh, C.P., Pinto-Plata, V., Celli, B., Marchetti, N., Criner, G., Bueno, R., Washko, G., et al. (2018). Human Lung DNA Methylation Quantitative Trait Loci Colocalize with Chronic Obstructive Pulmonary Disease Genome-Wide Association Loci. Am. J. Respir. Crit. Care Med. *197*, 1275–1284.

The American Journal of Human Genetics, Volume *106*

Supplemental Data

Smoking, DNA Methylation, and Lung Function: a Mendelian Randomization Analysis to Investigate Causal Pathways

Emily Jamieson, Roxanna Korologou-Linden, Robyn E. Wootton, Anna L. Guyatt, Thomas Battram, Kimberley Burrows, Tom R. Gaunt, Martin D. Tobin, Marcus Munafò, George Davey Smith, Kate Tilling, Caroline Relton, Tom G. Richardson, and Rebecca C. Richmond

Supplementary figures

Figure S1. Quantile-quantile plot of the observed vs expected p-values of the associations between smoking-associated DNA methylation and lung function (FEV_1).

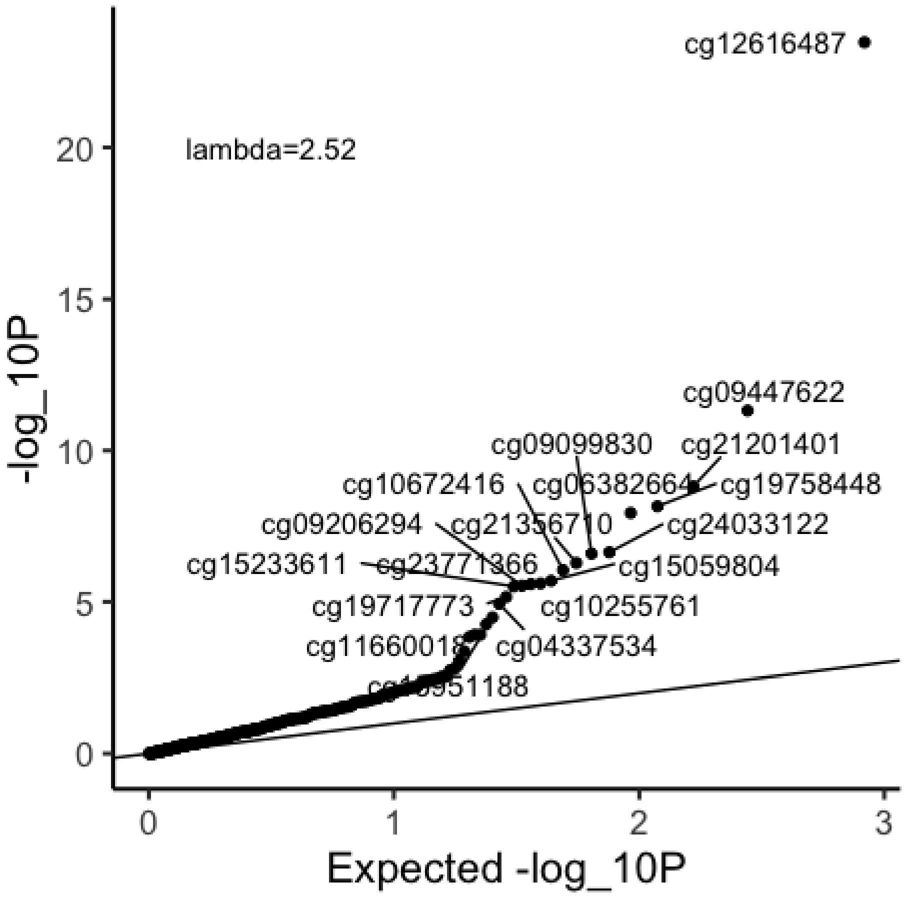

The line represents the null hypothesis of no association with lung function. Deviation from the expected distribution of P-values is evident with a lambda of 2.52.

Figure S2. Results of MR analysis of the effect of smoking-associated DNA methylation on lung function (FEV1) stratified by smoking status.

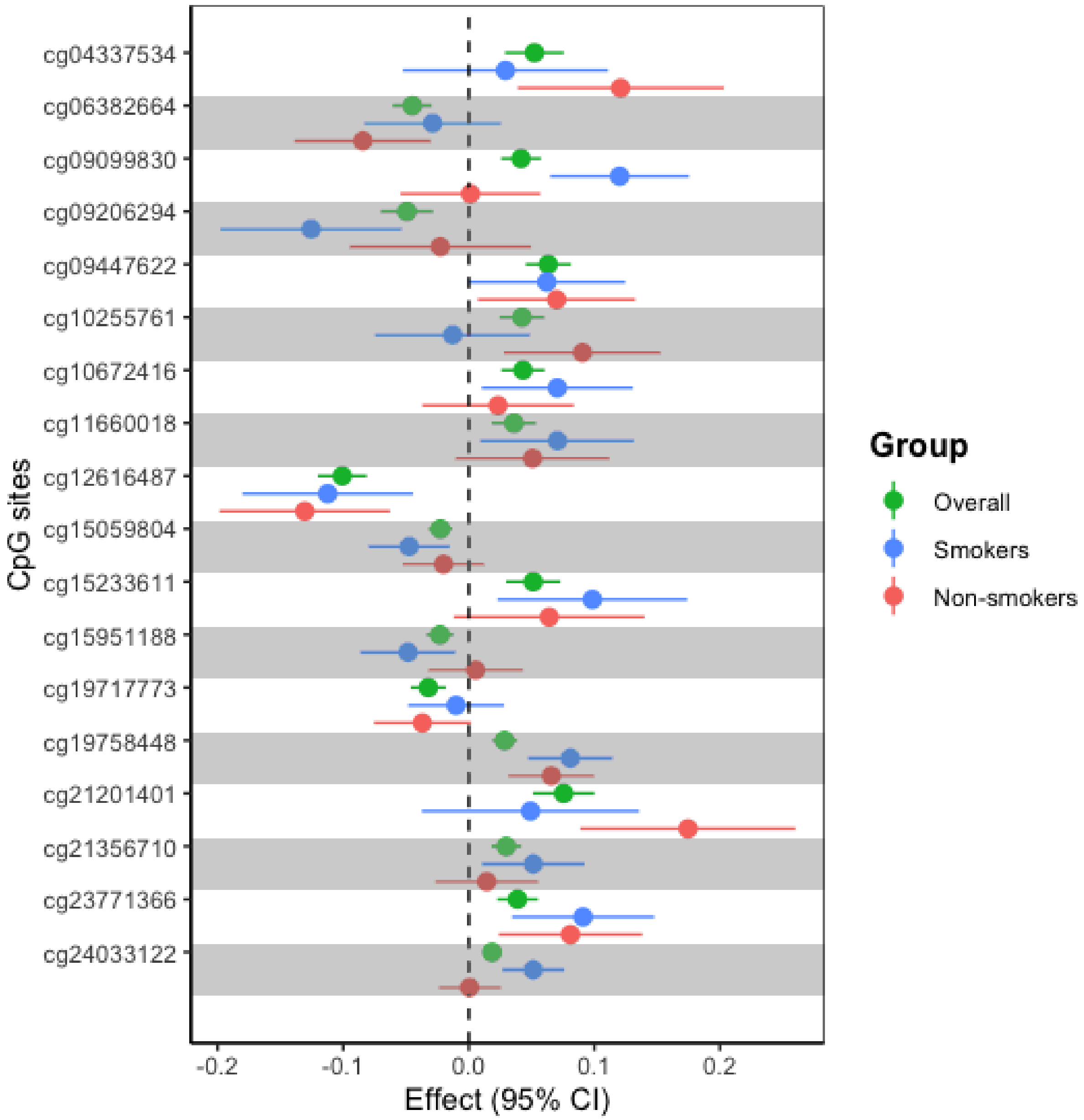

Effect sizes and 95% confidence intervals (CI) for each of the top eighteen CpG sites are shown for the overall UK Biobank sample in green, and the smoking and non-smoking UK BiLEVE samples in blue and red, respectively.

Figure S3. Results of MR analysis of the effect of smoking-associated DNA methylation on lung function (FEV1, FVC and FEV1/FVC)

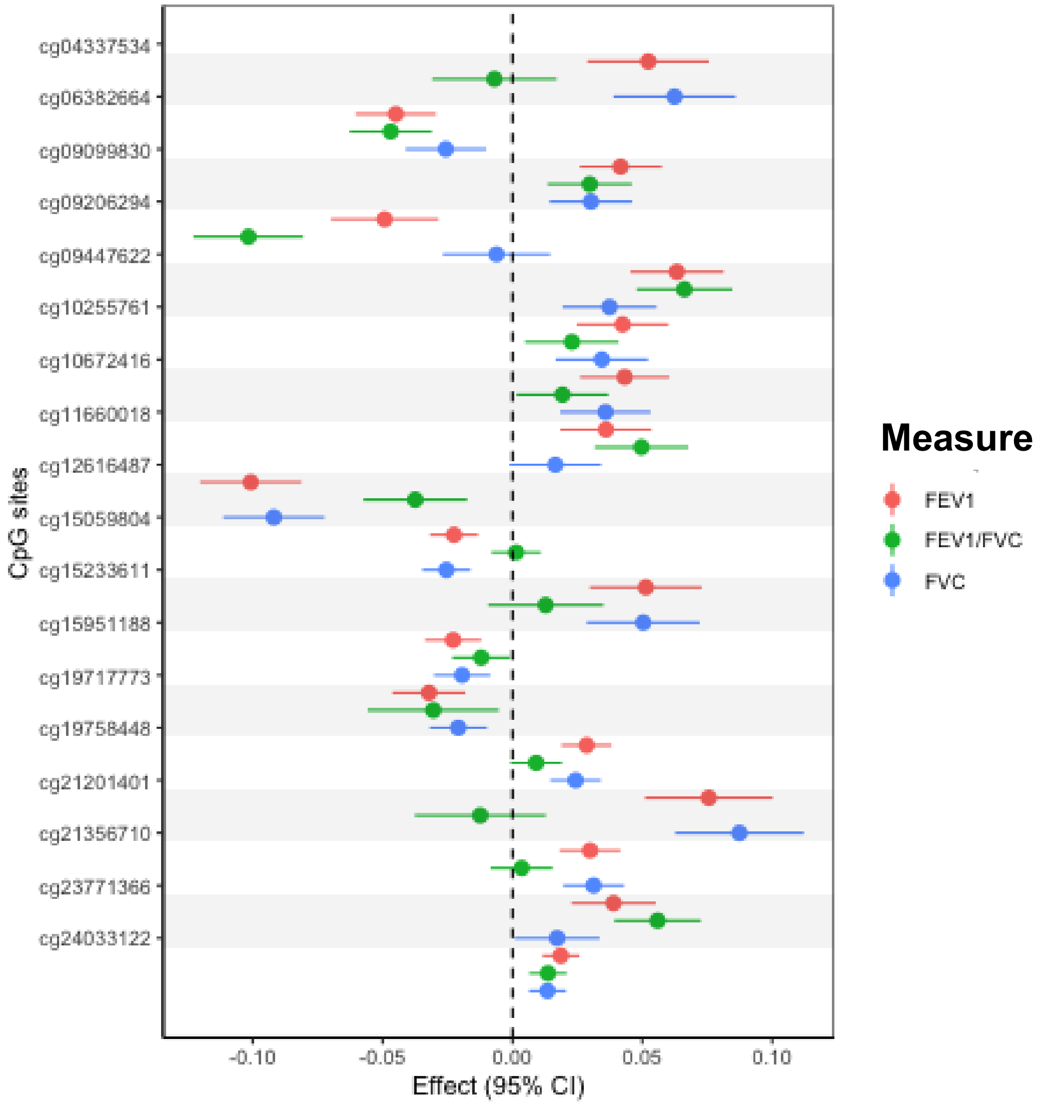

Effect sizes and 95% confidence intervals (CI) for each of the top eighteen CpG sites are shown for FEV1 in red, FEV1/FVC in green and FVC in blue.

Figure S4. Results of MR analysis of the effect of smoking-associated DNA methylation on lung disease (asthma and COPD)

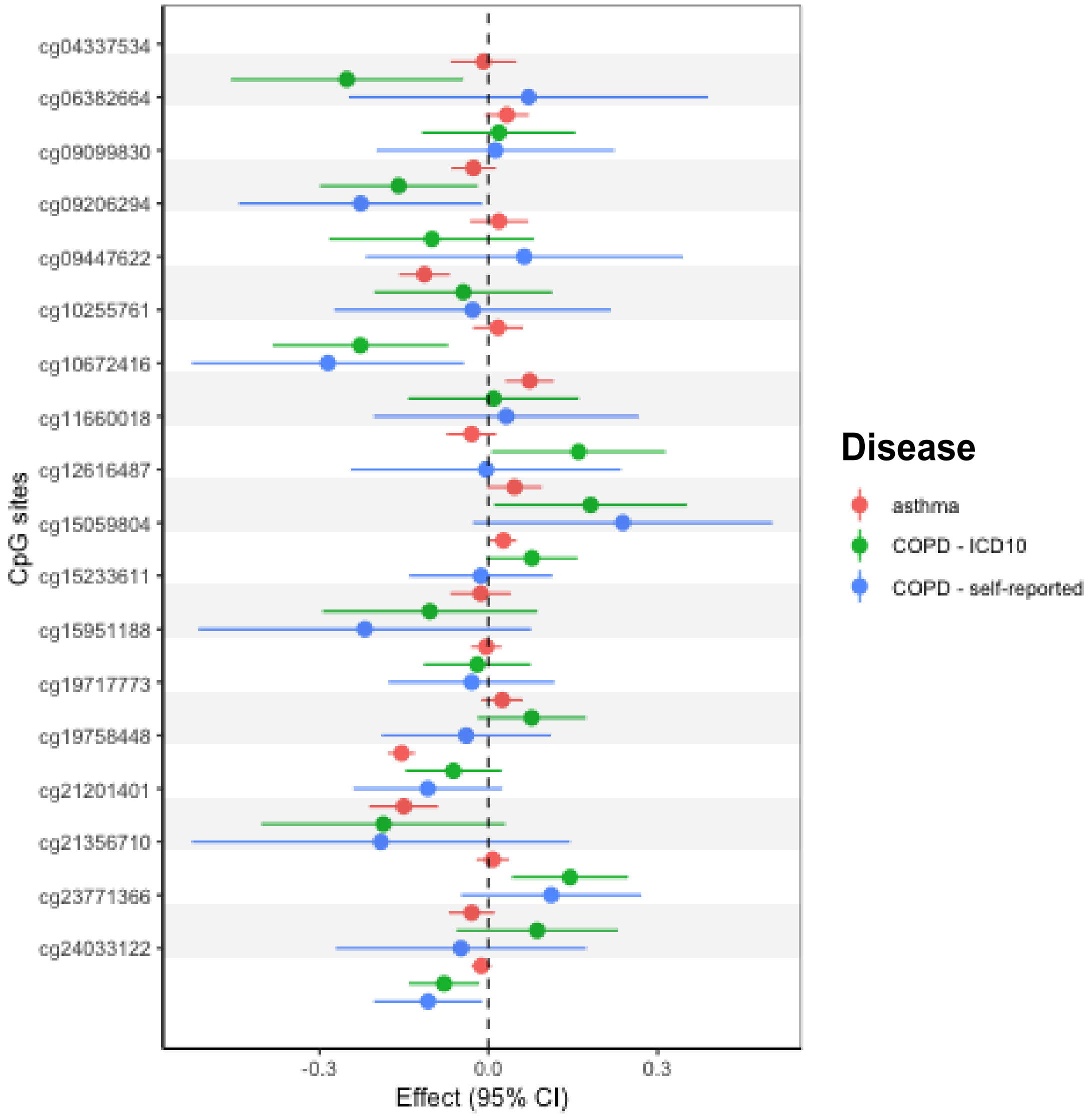

Effect sizes and 95% confidence intervals (CI) for each of the top eighteen CpG sites are shown for asthma in red, COPD obtained from ICD-10 codes in green and COPD based on self-report in blue

Figure S5. Comparison of observational and Mendelian randomization effect estimates of smoking on DNA methylation at smoking-related CpG sites*

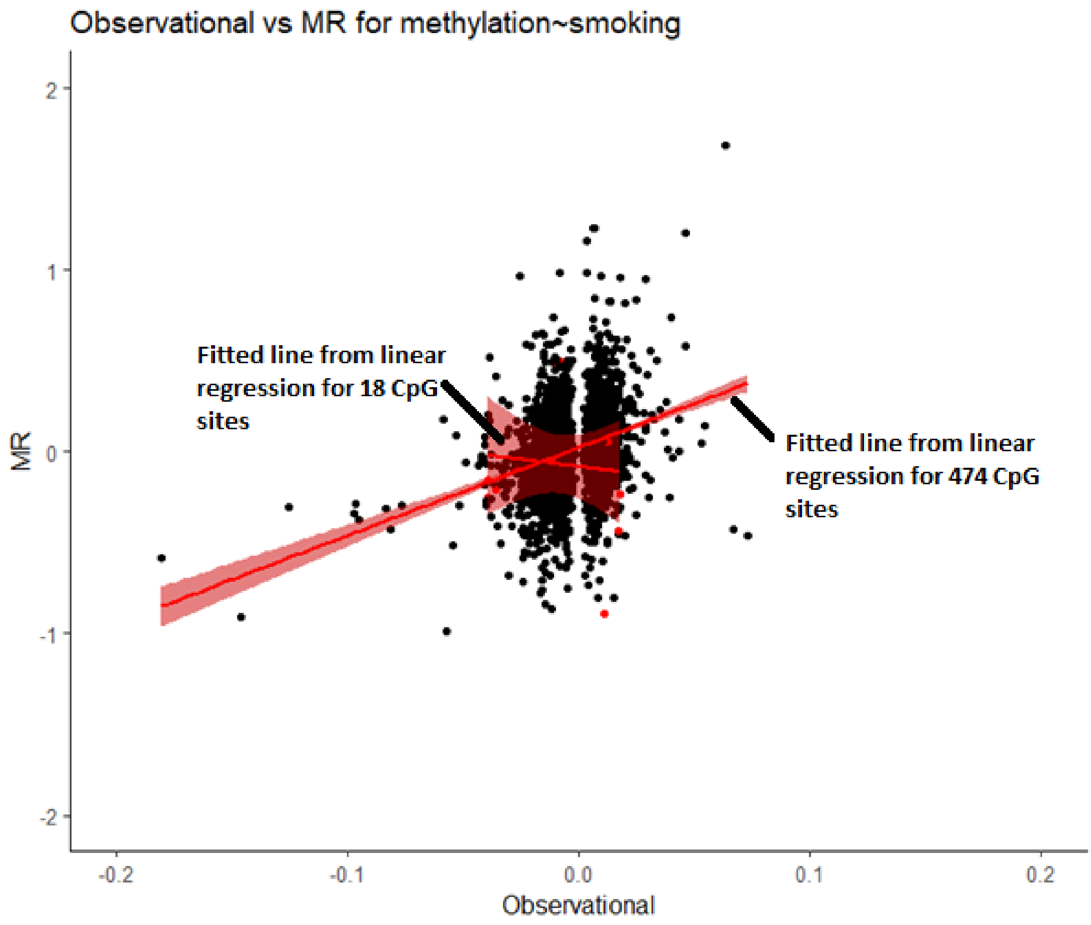

*Obtained from epigenome-wide association study of smoking by Joehanes et al, 2016 [1]

1 Joehanes, R. *et al.* Epigenetic Signatures of Cigarette Smoking. *Circ.-Cardiovasc. Genet.* **9**, 436-447, doi:10.1161/circgenetics.116.001506 (2016).

Figure S6. Comparison of MR analyses accounting for correlated mQTLs with those using only independent mQTLs

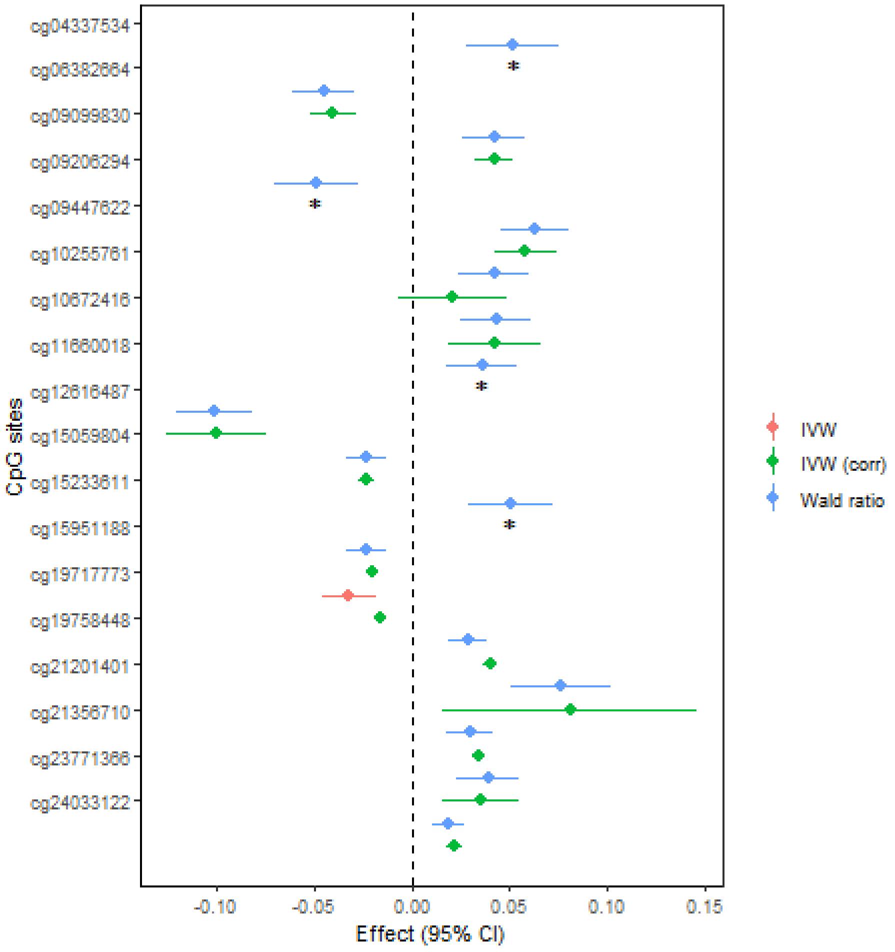

Effect sizes and 95% confidence intervals (CI) for each of the top eighteen CpG sites are shown from MR using only independent mQTLs (IVW in red and Wald ratio in blue), and accounting for correlated mQTLs (IVW (corr) in green). CpG sites where there was only one mQTL in the correlation analysis are indicated with an asterisk.

Figure S7. Egger regression plots for MR analysis accounting for correlated mQTLs

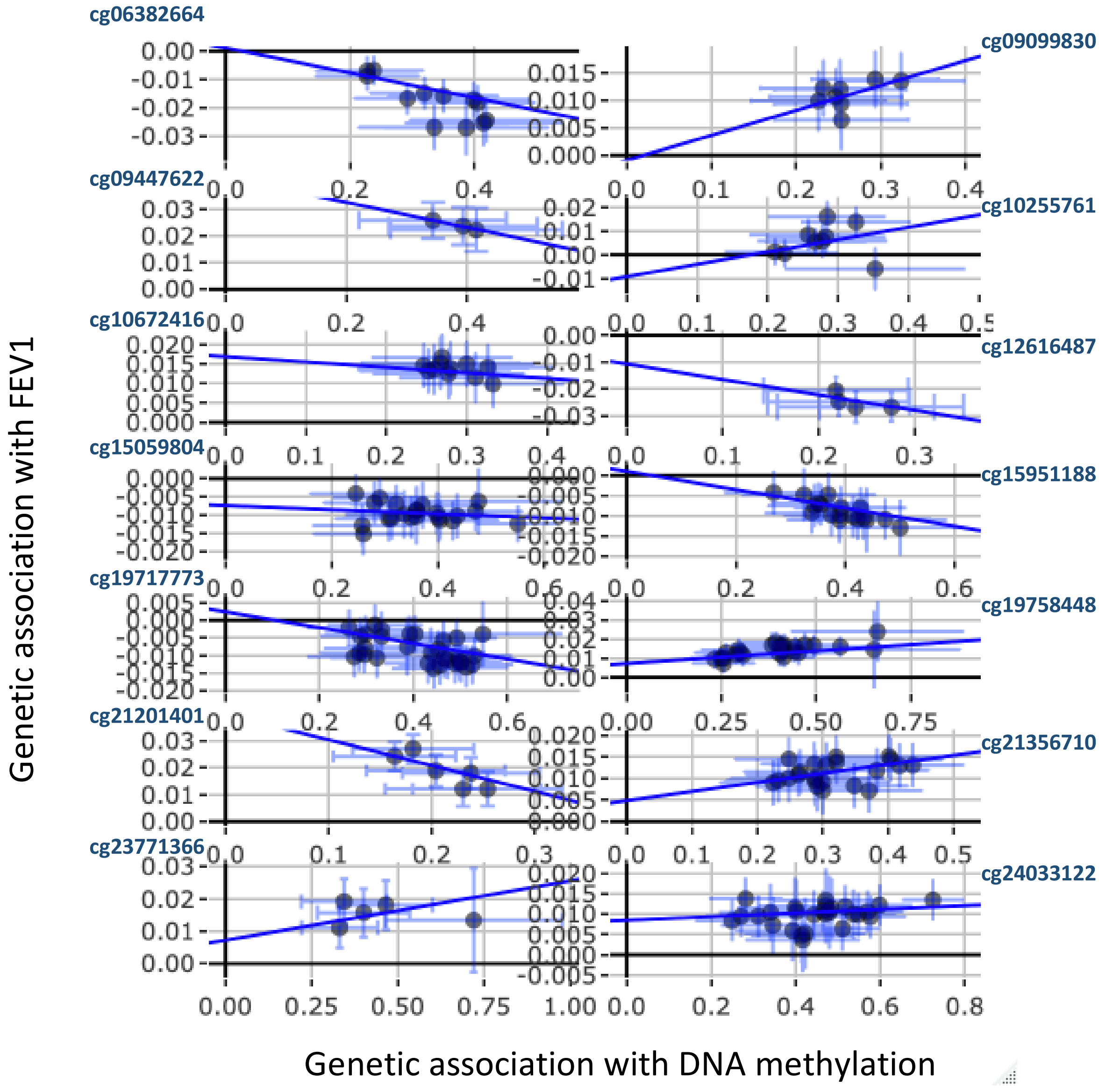

Where MR Egger regression line intercept deviates from the origin, this is indicative of directional pleiotropy

Figure S8. Comparison of MR analyses accounting for correlated mQTLs

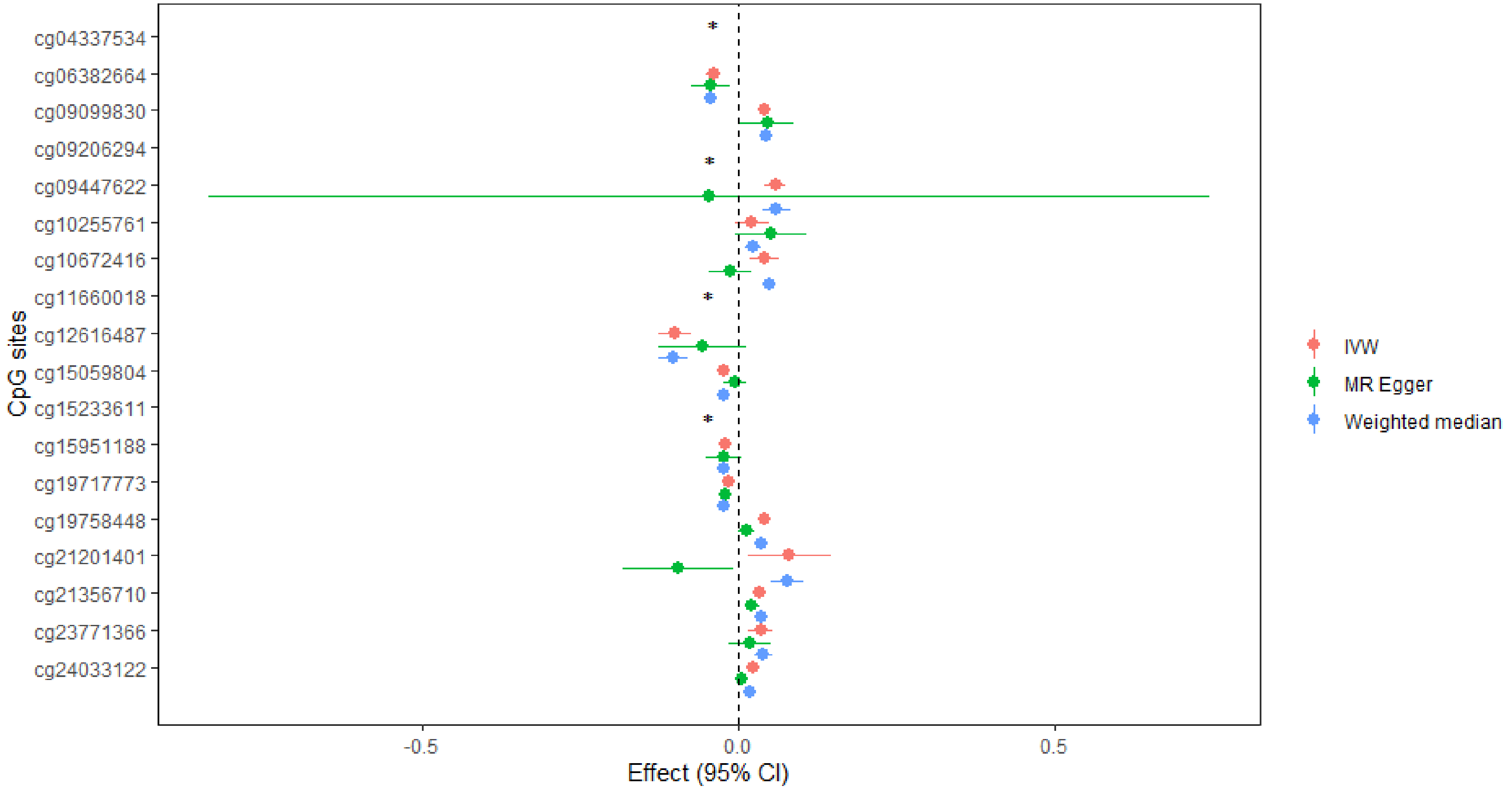

Effect sizes and 95% confidence intervals (CI) for each of the top eighteen CpG sites are shown from MR IVW in red, MR Egger in green and the weighted median approach in blue. Sites where there were too few mQTLs to perform the analysis are indicated with an asterisk.

Figure S9. Locus zoom plots for multiple-trait colocalization analysis

a) cg15951188

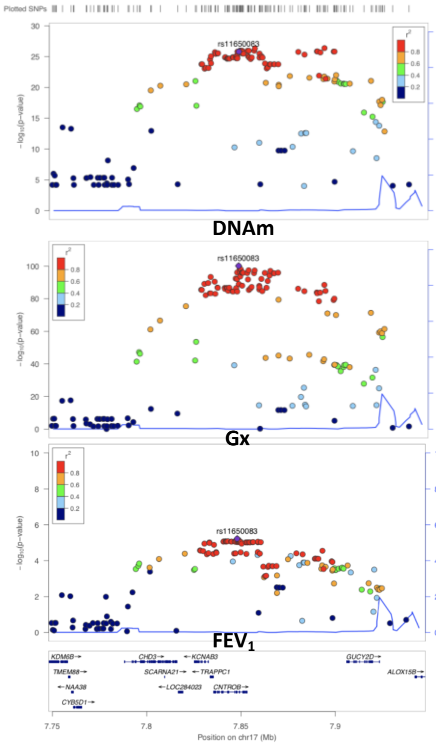

b) cg12616487

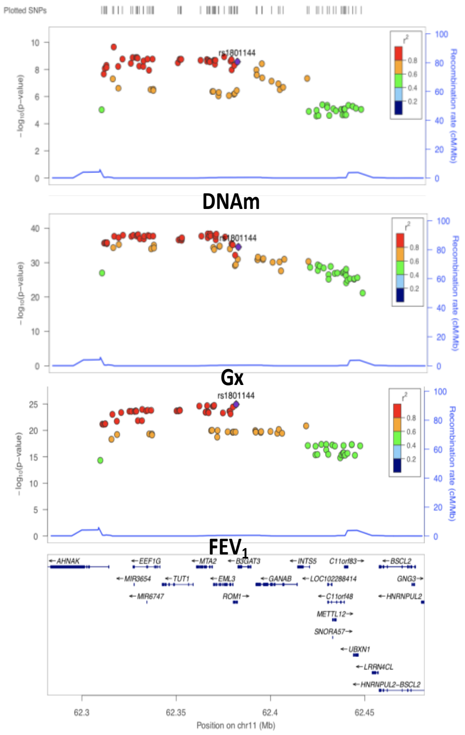

c) cg21201401

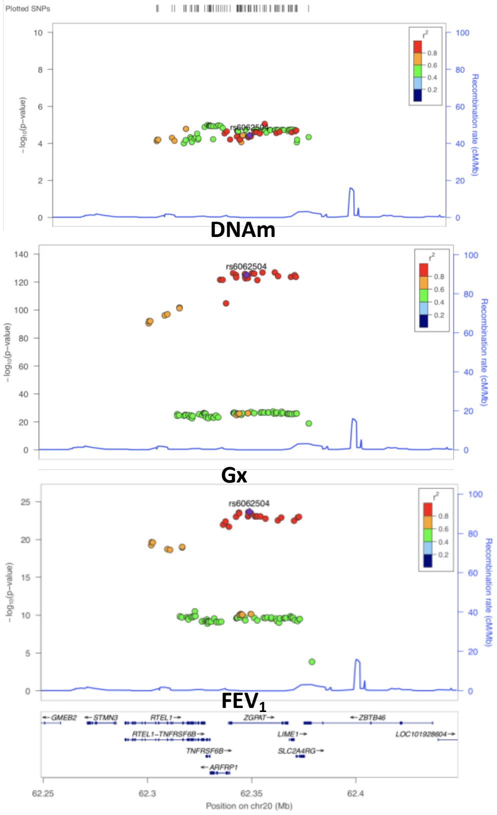

d) cg15233611

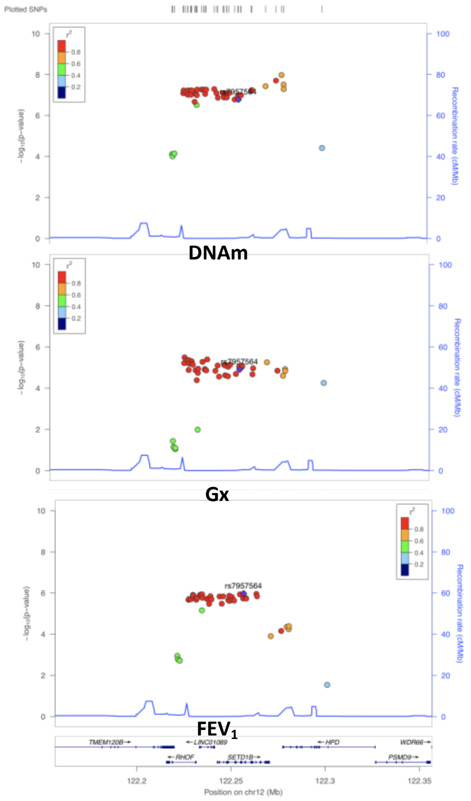

e) cg04337534

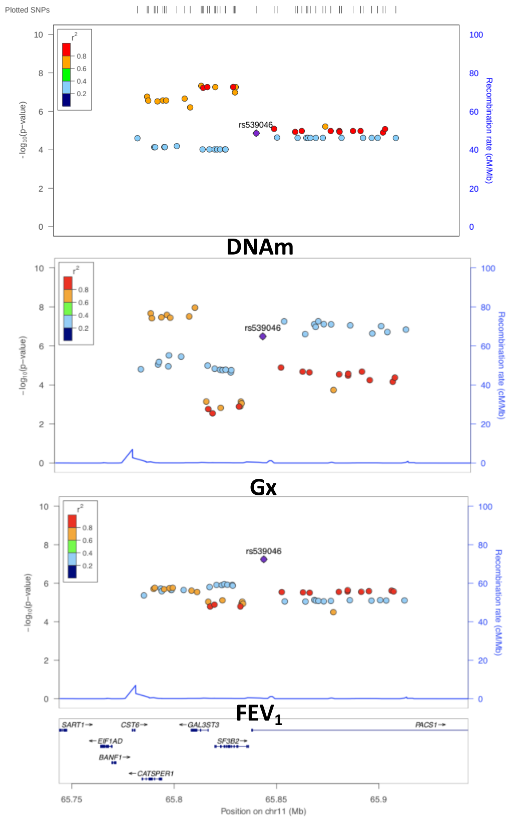

f) cg09447622

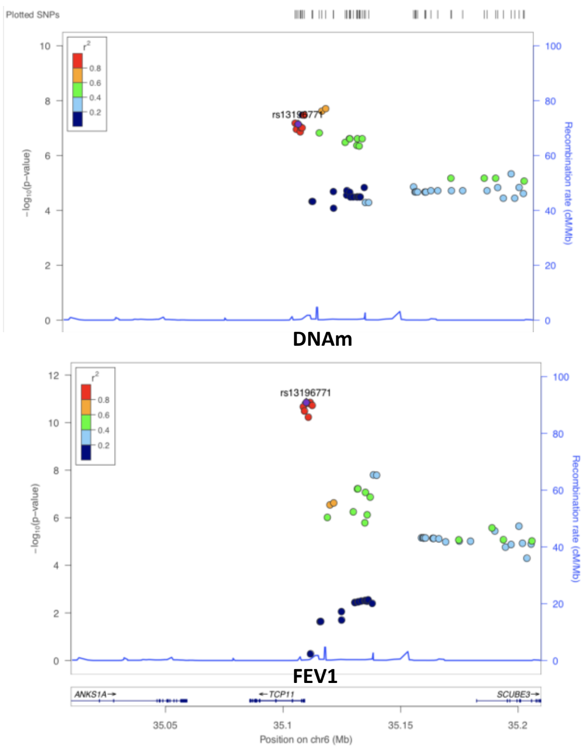

g) cg19758448

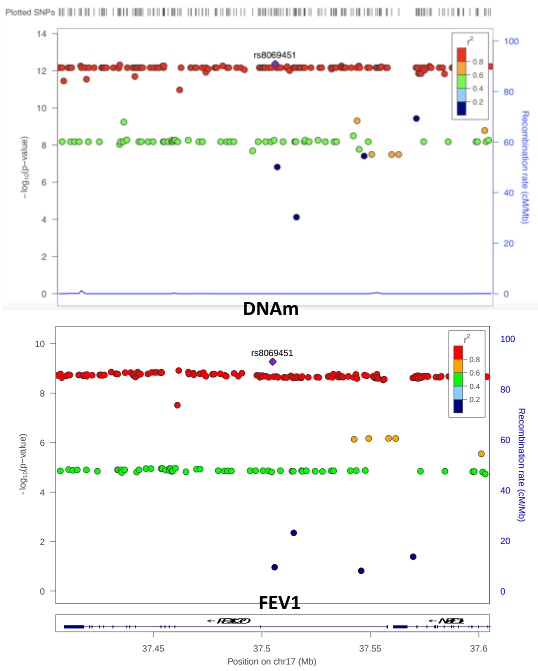

h) cg09206294

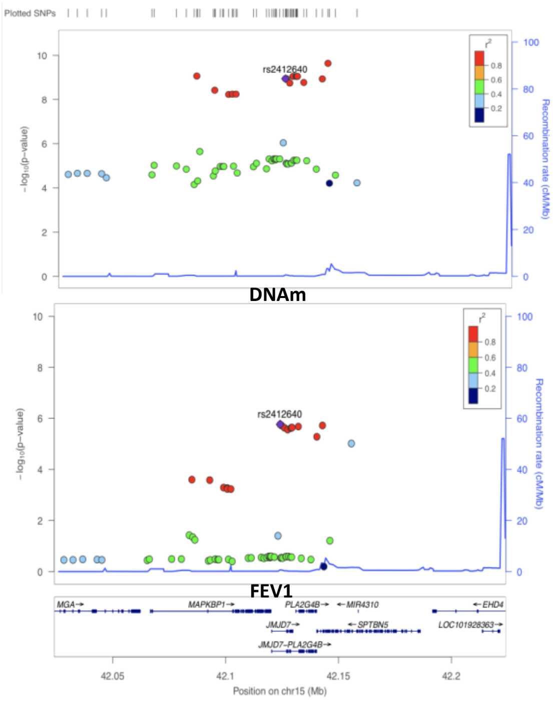

i) cg21356710

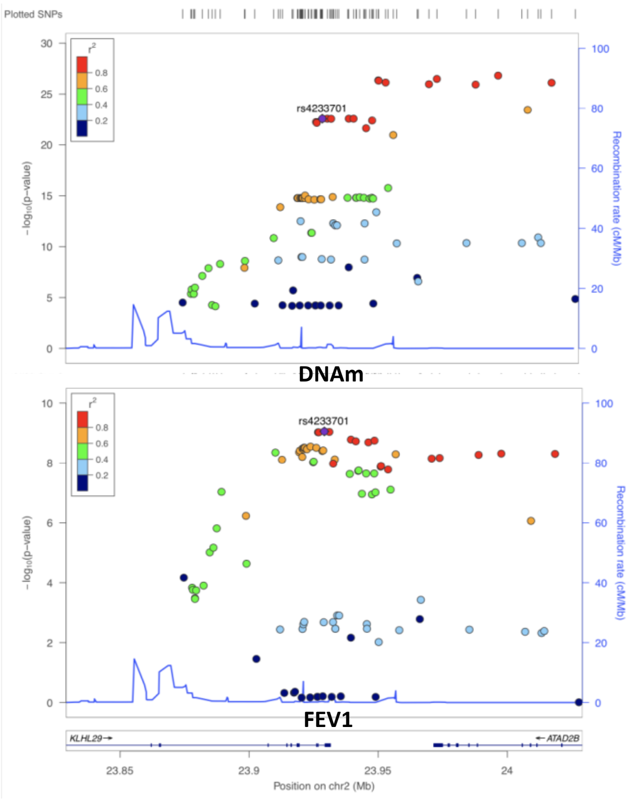

DNAm = DNA methylation; Gx = Gene expression; FEV1 = forced expiratory volume in 1 second
